# COVID-19 Booster Vaccine Messaging in Emergency Departments: A Cluster Randomized Clinical Trial

**DOI:** 10.1001/jamanetworkopen.2025.37655

**Published:** 2025-10-15

**Authors:** Melanie F. Molina, Stephanie A. Eucker, Kristin L. Rising, Efrat R. Kean, Zubaid Rafique, Heba Mesbah, David V. Glidden, Mireya I. Arreguin, Christopher Alvarez, Robert M. Rodriguez

**Affiliations:** 1 Department of Emergency Medicine, University of California, San Francisco; 2 Department of Emergency Medicine, Duke University, Durham, North Carolina; 3 Department of Emergency Medicine, Sidney Kimmel Medical College, Thomas Jefferson University, Philadelphia, Pennsylvania; 4 Center for Connected Care, Thomas Jefferson University, Philadelphia, Pennsylvania; 5 Henry JN Taub Department of Emergency Medicine, Baylor College of Medicine, Houston, Texas; 6 Department of Epidemiology & Biostatistics, University of California, San Francisco; 7 Department of Internal Medicine, University of California, Riverside

## Abstract

**Importance:**

Patient uptake of updated COVID-19 vaccines is crucial for reducing severe outcomes, yet national uptake remains low.

**Objective:**

To determine if tailored messaging or simple inquiry about vaccine acceptance increases 30-day uptake of updated COVID-19 vaccines among emergency department (ED) patients.

**Design, Setting, and Participants:**

This 3-arm, cluster randomized clinical trial conducted from January 29 to June 18, 2024, enrolled adult ED patients who had not received a COVID-19 vaccine in the prior 6 months across 6 EDs in San Francisco, California; Philadelphia, Pennsylvania; Houston, Texas; and Durham, North Carolina.

**Interventions:**

Intervention M involved tailored messaging about updated COVID-19 vaccines and inquiry about vaccine acceptance. Intervention Q involved only inquiry about vaccine acceptance. Usual care (no messaging or vaccine acceptance questions) served as the control.

**Main Outcomes and Measures:**

The primary outcome was updated COVID-19 vaccine receipt within 30 days of the ED visit, assessed via electronic health record review and follow-up telephone calls. Secondary outcomes included vaccine acceptance and vaccination during the ED visit. Outcomes were also compared between study sites that had the updated COVID-19 vaccine available and those that did not.

**Results:**

Of 852 participants (median age, 47 years [IQR, 33-63 years]; 464 [54.5%] women), 247 (29.0%) were in the intervention M group, 273 (32.0%) in the intervention Q group, and 332 (39.0%) in the control group. Vaccine uptake at 30 days was not significantly higher in either the intervention M group compared with control (14 participants [5.7%] vs 10 [3.0%]; absolute difference, 2.7 percentage points [pp] [95% CI, −0.8 to 6.3 pp]) or the intervention Q group compared with control (11 [4.0%] vs 10 [3.0%]; absolute difference, 1.0 pp [95% CI, −2.0 to 4.2 pp]). However, at sites where vaccines were available, the intervention M group had higher uptake compared with control (13 of 132 [9.8%] vs 5 of 150 [3.3%]; absolute difference, 6.5 pp [95% CI, 0.5-12.5 pp]).

**Conclusions and Relevance:**

In this cluster randomized clinical trial of adult ED patients, tailored messaging and simple inquiry alone did not significantly increase 30-day updated COVID-19 vaccine uptake among patients. The slight increase in uptake among participants in the EDs that had access to the updated COVID-19 vaccines suggested that vaccine availability was an effect modifier, underscoring the importance of opportunity and convenience in vaccine delivery.

**Trial Registration:**

ClinicalTrials.gov Identifier: NCT06156215

## Key Points

**Question:**

Do tailored messaging interventions about updated COVID-19 vaccines and simple inquiry about vaccine acceptance increase vaccine uptake among emergency department (ED) patients within 30 days of the ED visit?

**Findings:**

In this 3-arm, cluster randomized clinical trial of 852 participants across 6 EDs, neither a tailored messaging intervention nor inquiry about vaccine acceptance significantly increased 30-day vaccine uptake compared with usual care.

**Meaning:**

These findings suggest that tailored messaging and simple inquiry alone may be insufficient to increase updated COVID-19 vaccine uptake among ED patients.

## Teaser

This cluster randomized clinical trial examines whether tailored messaging about updated COVID-19 vaccines and simple inquiry about vaccine acceptance during an emergency department visit increase patients’ vaccine uptake within 30 days.

## Introduction

With evidence of continued benefit from updated (booster) COVID-19 vaccines,^1,2^ especially among older adults and immunocompromised populations,^2^ as of 2024, the Centers for Disease Control and Prevention (CDC) had recommended individuals aged 6 months or older receive 2024-2025 updated vaccines, with those aged 65 years or older recommended to receive 2 doses.^3,4^ Despite these recommendations, only 17.4% of US adults had received an updated vaccine as of early November 2024.^5^ Our group’s prior work examining hesitancy toward receiving updated COVID-19 vaccines among emergency department (ED) populations revealed a lack of opportunity, a need for more information, concerns about adverse effects, and the belief that an updated vaccine was unnecessary after the initial series as primary drivers of decreased uptake.^6^

Our group previously demonstrated that messaging for the initial COVID-19 series and influenza vaccines resulted in greater 30-day uptake among ED patients.^7,8^ With $78 billion in COVID-19 vaccine procurement and distribution during the pandemic,^9,10^ vaccines were freely available at all participating EDs of our group’s original COVID-19 vaccine messaging study.^7^ However, in May 2023, the COVID-19 public health emergency ended,^11^ and the costs for vaccines shifted to health insurers and patients via commercial payment structures.^12^ Without federally sponsored vaccine allocations, hospitals and other health care institutions faced 2 choices: forgo providing updated COVID-19 vaccines or source vaccines through standard commercial mechanisms.

Given the low uptake of updated COVID-19 vaccines, potentially exacerbated by decreased vaccine availability and cost shifting, we sought to determine whether messaging platforms with information about updated COVID-19 vaccines would lead to increased uptake among underserved ED populations, as it did in our group’s previous vaccine messaging trials.^7,8^ We conducted a randomized clinical trial (RCT) to determine whether (1) updated COVID-19 vaccine messaging and (2) asking ED patients whether they would accept an updated COVID-19 vaccine without messaging would increase 30-day uptake among ED patients compared with usual care (no messaging or vaccine acceptance questions). As secondary aims, we sought to examine the potential modifying effects of vaccine availability in hospitals and vaccine-related charges on vaccine acceptance and uptake.^13^

## Methods

### Design

We conducted a 3-arm, cluster RCT (Promotion of Booster COVID-19 Vaccination in the Emergency Department, or PROBOOSTVAXED, trial) from January 29 to June 18, 2024, comparing 2 interventions with control (usual care) in 6 EDs at a mix of academic and safety-net hospitals in 4 US cities: San Francisco, California; Philadelphia, Pennsylvania; Houston, Texas; and Durham, North Carolina. The University of California, San Francisco Committee on Human Research approved all trial procedures as a central institutional review board with multisite reliance mechanisms at the other sites. All participants provided verbal consent for study participation and signed a Health Insurance Portability and Accountability Act release form for record review. Written informed consent was obtained if participants agreed to a follow-up telephone call. We followed the Consolidated Standards of Reporting Trials (CONSORT) reporting guideline. The trial protocol is outlined in Supplement 1.

### Population

We enrolled ED patients during five 6- to 8-hour daytime blocks per week (9 am to 5 pm Monday-Friday). Research personnel reviewed ED dashboards to identify eligible patients and queried clinicians and patients regarding inclusion and exclusion criteria. We included adults who were (1) not vaccinated for COVID-19 within the prior 6 months (self-report), (2) able to provide written informed consent, (3) fluent in English or Spanish, and (4) able to complete study interventions in the ED (ie, watch a 3-minute video clip). We excluded patients who (1) were unable to complete a survey because of major trauma, intoxication, altered mental status, or critical illness; (2) were in police custody or incarcerated; (3) had a psychiatric chief complaint or hold; (4) had a medical reason not to receive a COVID-19 vaccine; or (5) were suspected of having acute COVID-19 illness. Criteria 4 and 5 were at the discretion of the patient’s treating clinician. We withdrew patients from the trial who became too sick to continue participation, had a change in mental status, left the ED suddenly without notifying clinicians, or withdrew consent during data collection.

### Randomization

The unit of randomization was single days, in which all participants were given usual care (control), intervention Q (acceptance question), or intervention M (updated COVID-19 vaccine messaging and acceptance question). We developed a 135-day trial calendar using pseudorandom numbers to permute days within 9 blocks consisting of 15 consecutive weekdays at each site, such that each site would have 5 days of each arm randomly dispersed within the 15-day block (eTable 1 in Supplement 2).

### Procedures and Interventions

We conducted study procedures during patient waiting times in the ED and did not compensate patients for participation. After obtaining written consent, staff administered the intake survey to participants, which assessed demographic information (eMethods 1 in Supplement 2).

After the intake survey on intervention M days, research staff attempted to deliver 3 PROBOOSTVAXED messaging platforms in either English or Spanish (Box); these platforms were developed and validated from earlier qualitative work^14,15^ and adapted to updated COVID-19 vaccines based on previously reported reasons for hesitancy.^6^ Staff asked participants to watch a 3-minute audiovisual recording about the updated COVID-19 vaccines. If participants agreed, staff provided them with a link to a YouTube video to stream on their smartphone. If participants did not have a smartphone or streaming was unavailable, staff showed participants the video on an electronic tablet. After watching the video, staff provided an informational flyer about updated COVID-19 vaccines featuring physicians and personnel who matched participant-reported racial and ethnic characteristics, as race-concordant messages have been shown to increase desire for additional COVID-related information.^16^ After leaving the room, research staff gave 1 of the participant’s ED clinicians a scripted message regarding updated COVID-19 vaccines to deliver orally to the participant by directly reading the message or paraphrasing in their own words.

Box. **Updated COVID-19 Vaccine Messaging Platforms Composing Intervention M**

A 3-minute public service announcement–type video (5 versions, all with the same wording in the message but each with a different pair of physician messengers):
  - Black physicians
  - Latinx physicians, English version
  - Latinx physicians, Spanish version
  - Multiracial physicians
  - White physicians
A single-page information flyer (5 versions, all with the same format and captions but each with different pictures of patients receiving the vaccine and health care clinicians administering the vaccines):
  - Predominantly Black patients and clinicians
  - Predominantly Latinx patients and clinicians, English version
  - Predominantly Latinx patients and clinicians, Spanish version
  - Multiracial patients and clinicians
  - Predominantly White patients and clinicians
A 7-sentence script for COVID-19 booster vaccine messaging (from an emergency department physician or nurse) on a sheet of paper

Following delivery of the messaging platforms, staff administered the vaccine acceptance survey (eMethods 2 in Supplement 2), in which participants were asked, “*Would you accept the COVID booster vaccine in the emergency department today if your doctor or provider offered it to you?*” If participants answered yes, staff read an additional statement acknowledging the potential vaccine cost: “*If you receive a vaccine in the ED today, you may be responsible for the cost, unless it is covered by your insurance.”* They were then asked, “*Would you still accept the COVID booster vaccine in the emergency department today if your doctor or provider offered it to you?*”

On intervention Q days, after the intake survey was completed, staff administered the vaccine acceptance survey followed by the vaccine acceptance questions without any messaging (eMethods 2 in Supplement 2). On control days, staff did not provide messaging or ask the vaccine acceptance questions. Although participants on intervention Q and control days did not receive messaging, we did not prevent the delivery of other COVID-19 vaccine messaging or questions from clinicians.

To assess outcomes, research staff who were blinded to study arms conducted electronic health record (EHR) review and telephone follow-up 28 to 32 days after the initial ED visit, with a maximum of 3 telephone call attempts. If staff were unable to reach participants via telephone and did not find evidence of 30-day vaccination within the EHR, it was assumed the participant did not receive an updated COVID-19 vaccine. As part of quality control, we reviewed cases in which participants stated they would accept the vaccine but did not receive it in the ED.

### Vaccine Availability and Clinician Notification

Investigators had no control over vaccine availability at study sites. In contrast to our group’s prior vaccine trials^7,8^ in which vaccines were directly available from all ED site pharmacies, in the current study, only 3 of the 6 sites had the updated COVID-19 vaccine available in their hospitals, and none of the sites had it directly available from their ED pharmacies. At the sites where the vaccine was available, research staff attempted to notify clinicians if participants answered they would accept the updated COVID-19 vaccine and granted permission to share this information with their clinician. The participant’s clinician could then order the vaccine from the hospital’s central pharmacy. If participants accepted the updated COVID-19 vaccine and the site did not have vaccine available, staff informed the participant that the vaccine was unavailable in the ED and provided them with a list of nearby sites where they could obtain it (eFigure in Supplement 2).

### Study Outcomes

The primary outcome was receipt of an updated COVID-19 vaccine within 30 days of the initial (index) ED visit, comparing the intervention M group with the control group. Secondary outcomes were (1) updated vaccine receipt within 30 days of the initial ED visit, comparing the intervention Q group with the control group and the intervention M group with the intervention Q group; (2) updated vaccine receipt during the index ED visit, ascertained by EHR review, comparing the intervention M group with control, intervention Q group with control, and intervention M vs intervention Q groups; and (3) initial updated vaccine acceptance, defined as a “yes” response to the ED vaccine acceptance question prior to the statement on potential incurred cost, comparing the intervention M and intervention Q groups. In addition, as an explicit aim, we sought to compare 30-day uptake between study sites that had the updated COVID-19 vaccine available and those that did not.

### Sample Size

For our a priori sample size calculation, we used a clinically meaningful effect size (an increase in vaccine uptake of 7 percentage points [pp]) and baseline (control group) 30-day vaccine uptake rates of 5 pp derived from our group’s previous research.^7^ We calculated a sample size of 248 participants per arm (744 total) to detect a difference of 7 pp when comparing the intervention M period with control or the intervention Q period with control with 80% power on a 2-sided, 0.05-level test.

### Statistical Analysis

We analyzed outcomes according to the study arms to which patients were allocated (intention-to-treat analysis). We modeled primary and secondary outcomes using mixed-effects logistic regression with a random-center effect to accommodate potential within-center characteristics, as well as a term for randomized intervention with risk differences.^17^ However, the lower-than-expected number of observed outcomes prevented the convergence of mixed models, and the random effect was not included in our analyses.

We summed vaccine acceptance question responses in the intervention M and intervention Q groups before and after the cost statement to understand how cost might impact vaccine acceptance, presenting the differences as percentage points with 95% CIs. We conducted preplanned analyses of patient subgroups, stratifying according to the following characteristics: age in tertiles, gender, self-reported race (defined by the participant), self-reported Latinx ethnicity, having primary care, and primary language. Race and ethnicity were assessed to investigate disparities in vaccine uptake. Categories were American Indian or Alaska Native; Asian; Black; Latinx; Middle Eastern; Native Hawaiian or Pacific Islander; White, non-Latinx; and other (included several Latin American and Asian nationalities, multiracial, and unknown). Subgroup differences were considered statistically significant if the omnibus test of treatment by subgroup interaction was significant at 2-sided *P* < .05. We managed data using REDCap^18^ and analyzed data using Stata, version 18 (StataCorp LLC).

## Results

### Study Participants

We screened 2532 patients across the 6 sites. Of these, 318 stated they had already received the updated vaccine, and 260 had other exclusions. Of the remaining 1954 patients, 919 agreed to participate; 67 were withdrawn for prespecified reasons, resulting in 852 trial participants (247 [29.0%] on intervention M days, 273 [32.0%] on intervention Q days, and 332 [39.0%] on control days) (Figure). Participants’ median age was 47 years (IQR, 33-63 years); 379 (44.5%) identified as men, 464 (54.5%) as women, and 9 (1.1%) as other gender. Five participants (0.6%) were American Indian or Alaska Native; 48 (5.6%), Asian; 287 (33.7%), Black; 170 (20.0%), Latinx; none, Middle Eastern; 12 (1.4%), Native Hawaiian or Pacific Islander; 345 (40.5%), White, non-Latinx; and 24 (2.8%), other race and ethnicity. A total of 98 (11.5%) lacked health insurance, 187 (21.9%) lacked primary care, 72 (8.5%) spoke Spanish primarily, and 70 (8.2%) were unhoused or marginally housed (Table 1).

**Figure.**
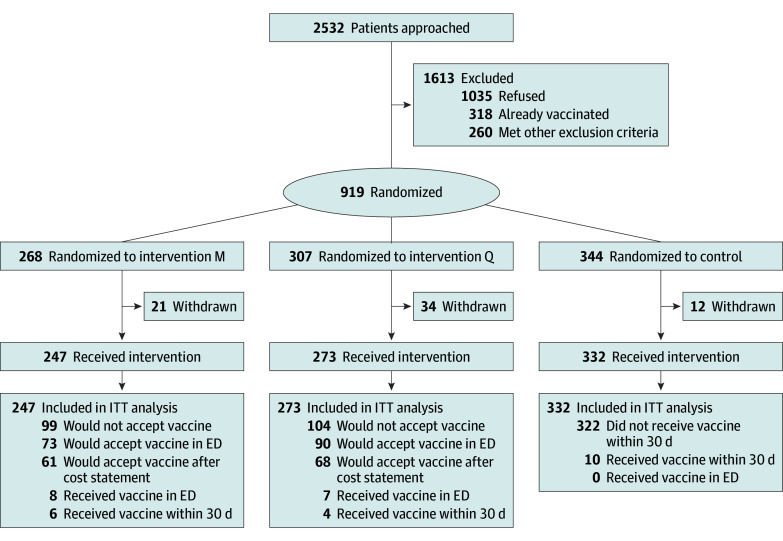
CONSORT Flow Diagram ED indicates emergency department; ITT, intention to treat.

**Table 1.** Participant Characteristics

Characteristic	Participants^a^			
	All (N = 852)	Control (n = 332)	Intervention M (n = 247)	Intervention Q (n = 273)
Age, median (IQR), y	47 (33-63)	46 (33-61)	46 (33-62)	49 (33-61)
Gender				
Men	379 (44.5)	143 (43.1)	107 (43.3)	129 (47.3)
Women	464 (54.5)	186 (56.0)	136 (55.1)	142 (52.0)
Other^b^	9 (1.1)	3 (0.9)	4 (1.6)	2 (0.7)
Race and ethnicity^c^				
American Indian or Alaska Native	5 (0.6)	2 (0.6)	3 (1.2)	0
Asian	48 (5.6)	16 (4.8)	12 (4.9)	20 (7.3)
Black	287 (33.7)	119 (35.8)	83 (33.6)	85 (31.1)
Latinx	170 (20.0)	61 (18.4)	48 (19.4)	61 (22.3)
Middle Eastern	0	0	0	0
Native Hawaiian or Pacific Islander	12 (1.4)	4 (1.2)	4 (1.6)	4 (1.5)
White, non-Latinx	345 (40.5)	137 (41.3)	101 (40.9)	107 (39.2)
Other^d^	24 (2.8)	10 (3.0)	6 (2.4)	8 (2.9)
Health insurance				
City-based health care service	13 (1.5)	3 (0.9)	6 (2.4)	4 (1.5)
HMO or Kaiser	3 (0.4)	1 (0.3)	0	2 (0.7)
Medicaid	178 (20.9)	71 (21.4)	50 (20.2)	57 (20.9)
Medicare	263 (30.9)	101 (30.4)	84 (34.0)	78 (28.6)
None	98 (11.5)	34 (10.2)	34 (13.8)	30 (11.0)
Affordable Care Act	1 (0.1)	1 (0.3)	0	0
Private	344 (40.4)	139 (41.9)	82 (33.2)	123 (45.1)
Veterans Health Administration	11 (1.3)	3 (0.9)	5 (2.0)	3 (1.1)
Has a primary care clinic or physician				
Yes	661 (77.6)	264 (79.5)	188 (76.1)	209 (76.6)
No	187 (21.9)	68 (20.5)	56 (22.7)	63 (23.1)
Unsure	3 (0.4)	0	2 (0.8)	1 (0.4)
Primary language				
English	778 (91.3)	310 (93.4)	217 (87.9)	251 (91.9)
Spanish	72 (8.5)	20 (6.0)	30 (12.1)	22 (8.1)
Other^e^	2 (0.2)	2 (0.6)	0	0
Housing status				
Housed	781 (91.7)	302 (91.0)	226 (91.5)	253 (92.7)
Marginally housed	15 (1.8)	7 (2.1)	2 (0.8)	6 (2.2)
Unhoused	55 (6.5)	23 (6.9)	18 (7.3)	14 (5.1)

Abbreviation: HMO, health maintenance organization.

^a^ Data are presented as number (percentage) of participants unless otherwise indicated.

^b^ Included nonbinary, transgender men, transgender women, and preferred not to answer.

^c^ Participants were allowed to check all that applied; therefore, percentages sum to greater than 100.

^d^ Included several Latin American and Asian nationalities, multiracial, and unknown.

^e^ Included Chinese and unknown.

Of the 247 intervention M group participants, 194 (78.5%) said they watched the video, 211 (85.4%) said they viewed or read the flyer, and 61 (24.7%) said they received vaccine messaging from an ED clinician. Of the 273 intervention Q group participants, 4 (1.5%) stated they received vaccine messaging from an ED clinician.

### Primary and Secondary Outcomes

We attained 100% EHR follow-up. Of the 837 participants (98.2%) who did not receive the vaccine in the ED, 646 (77.2%) agreed to telephone follow-up, and we successfully reached 372 of those participants (57.6%). In our primary outcome analysis, the intervention M group did not have higher rates of 30-day COVID-19 vaccination than the control group (14 [5.7%] vs 10 [3.0%]: absolute difference, 2.7 pp; 95% CI, −0.8 to 6.3 pp) (Table 2).

**Table 2.** Updated COVID-19 Vaccine Acceptance and 30-Day Uptake

Vaccine acceptance	Participants, No. (%)			Difference in proportion, pp (95% CI)		
	Control (n = 332)	Intervention M (n = 247)	Intervention Q (n = 273)	Intervention M vs control	Intervention Q vs control	Intervention Q vs intervention M
Vaccinated within 30 d	10 (3.0)	14 (5.7)	11 (4.0)	2.7 (−0.8 to 6.3)	1.0 (−2.0 to 4.2)	−1.6 (−5.5 to 2.2)
Accepted in ED	0	73 (29.6)	90 (33.0)	29.6 (23.7 to 35.1)	33.0 (27.2 to 38.4)	3.4 (−4.6 to 11.3)
Accepted in ED after cost consideration	0	61 (24.7)	68 (24.9)	24.7 (19.2 to 30.0)	24.9 (19.6 to 29.9)	0.2 (−7.2 to 7.6)
Vaccinated in ED	0	8 (3.2)	7 (2.6)	3.2 (0.9 to 5.7)	2.6 (0.5 to 4.7)	−0.7 (−3.8 to 2.3)

Abbreviations: ED, emergency department; pp, percentage points.

Among sites that had the updated COVID-19 vaccine available for administration, the intervention M group had a greater 30-day vaccination rate than the control group (13 of 132 [9.8%] vs 5 of 150 [3.3%]; absolute difference, 6.5 pp [95% CI, 0.5-12.5 pp]), as well as a greater ED vaccination rate (8 of 132 [6.1%] vs 0 of 150; absolute difference, 6.1 pp [95% CI, 1.6-10.5 pp]). Similarly, the intervention Q group had a greater ED vaccination rate than control (7 of 130 [5.4%] vs 0 of 150; absolute difference, 5.4 pp [95% CI, 1.1-9.7 pp]) (eTable 2 in Supplement 2).

With respect to secondary outcomes, the intervention Q group and control group had similar 30-day vaccine uptake (11 [4.0%] vs 10 [3.0%]; absolute difference, 1.0 pp [95% CI, −2.0 to 4.2 pp]), as did the intervention M and intervention Q groups (14 [5.7%] vs 11 [4.0%]: absolute difference, −1.6 pp [95% CI, −5.5 to 2.2 pp]). All 3 groups had similar vaccine uptake during their index ED visit: intervention M, 8 (3.2%); intervention Q, 7 (2.6%); and control, 0. Combining participants in the intervention M and intervention Q groups (520 total) who received the vaccine acceptance questions, 163 (31.3%) accepted the vaccine initially, and 129 (24.8%) indicated they would still accept after the vaccine cost statement (absolute difference, 6.5 pp; 95% CI, 4.7-9.0 pp). Subgroup differences in 30-day vaccine uptake comparing intervention M, intervention Q, and control groups are presented in Table 3.

**Table 3.** Updated COVID-19 Vaccine 30-Day Uptake, Subgroup Analyses

Variable	All participants, No. (%) (N = 852)	Participants who received vaccine in 30 d, No./total No. (%)			Difference, pp (95% CI)		
		Control (n = 332)	Intervention M (n = 247)	Intervention Q (n = 273)	Intervention M vs control	Intervention Q vs control	Intervention Q vs intervention M
Age tertile, y							
18-38	296 (34.7)	1/115 (0.9)	3/92 (3.3)	1/89 (1.1)	2.4 (−2.2 to 7.3)	0.3 (−3.3 to 4.3)	−2.1 (−7.1 to 3.0)
39-57	287 (33.7)	3/111 (2.7)	3/79 (3.8)	4/97 (4.1)	1.1 (−4.4 to 7.2)	1.4 (−4.0 to 7.0)	0.3 (−6.3 to 6.5)
58-82	269 (31.6)	6/106 (5.7)	8/76 (10.5)	6/87 (6.9)	4.9 (−3.4 to 13.5)	1.2 (−5.9 to 8.7)	−3.6 (−12.7 to 5.4)
Gender							
Men	379 (44.5)	4/143 (2.8)	8/107 (7.5)	4/129 (3.1)	4.7 (−1.2 to 10.8)	0.3 (−4.1 to 4.8)	−4.4 (−10.6 to 1.7)
Women	464 (54.5)	6/186 (3.2)	5/136 (3.7)	6/142 (4.2)	0.5 (−3.7 to 5.0)	1.0 (−3.3 to 5.6)	0.5 (−4.4 to 5.4)
Race							
Asian, American Indian, or Pacific Islander^a^	61 (7.2)	0	2/18 (11.1)	1/22 (4.5)	11.1 (−7.1 to 28.4)	4.5 (−9.9 to 17.8)	−6.6 (−25.8 to 12.5)
Black	287 (33.7)	3/119 (2.5)	5/83 (6.0)	2/85 (2.4)	3.5 (−2.6 to 10.1)	−0.2 (−4.8 to 5.1)	−3.7 (−10.3 to 3.0)
White	357 (41.9)	6/143 (4.2)	5/102 (4.9)	5/112 (4.5)	0.7 (−4.7 to 6.6)	0.3 (−4.9 to 5.8)	−0.4 (−6.6 to 5.6)
Other^b^	147 (17.3)	1/49 (2.0)	2/44 (4.5)	3/54 (5.6)	2.5 (−6.3 to 11.5)	3.5 (−5.4 to 11.8)	1.0 (−9.2 to 10.4)
Latinx							
No	682 (80.0)	8/271 (3.0)	12/199 (6.0)	7/212 (3.3)	3.1 (−0.8 to 7.0)	0.3 (−2.9 to 3.8)	−2.7 (−7.0 to 1.5)
Yes	170 (20.0)	2/61 (3.3)	2/48 (4.2)	4/61 (6.6)	0.9 (−7.2 to 9.7)	3.3 (−5.3 to 11.7)	2.4 (−7.4 to 11.3)
Has primary care							
No	187 (21.9)	2/68 (2.9)	2/56 (3.6)	1/63 (1.6)	0.6 (−6.5 to 8.3)	−1.4 (−7.5 to 5.1)	−2.0 (−9.2 to 5.0)
Yes	661 (77.6)	8/264 (3.0)	11/188 (5.9)	10/209 (4.8)	2.8 (−1.2 to 7.0)	1.8 (−1.9 to 5.5)	−1.1 (−5.7 to 3.5)
Primary language							
English	778 (91.3)	10/310 (3.2)	11/217 (5.1)	10/251 (4.0)	1.8 (−1.7 to 5.6)	0.8 (−2.4 to 4.1)	−1.1 (−5.1 to 2.8)
Spanish	72 (8.5)	0	3/30 (10.0)	1/22 (4.5)	10.0 (−6.4 to 22.3)	4.5 (−10.3 to 17.9)	−5.5 (−20.1 to 11.8)
Vaccine available in ED							
No	440 (51.6)	5/182 (2.7)	1/115 (0.9)	3/143 (2.1)	−1.9 (−5.0 to 1.9)	−0.6 (−4.2 to 3.2)	1.2 (−2.5 to 4.6)
Yes	412 (48.4)	5/150 (3.3)	13/132 (9.8)	8/130 (6.2)	6.5 (0.5 to 12.5)	2.8 (−2.4 to 8.2)	−3.7 (−10.4 to 3.1)

Abbreviations: ED, emergency department; pp, percentage points.

^a^ Given small cell counts, these race categories were merged.

^b^ Included Latinx only, several Latin American and Asian nationalities, multiracial, and unknown.

## Discussion

In this 3-arm, cluster RCT at 6 high-volume EDs, neither tailored messaging regarding the updated COVID-19 vaccine (intervention M) nor the simple intervention of inquiring about updated vaccine acceptance (intervention Q) significantly increased 30-day vaccine uptake compared with usual care. However, among ED sites that had the updated vaccines available for administration, we found modest increases in 30-day uptake with intervention M and ED uptake with both interventions.

This study is the third in a series of studies evaluating the effectiveness of tailored messaging platforms on vaccine uptake in ED populations. The first study (PROCOVAXED), conducted in 2021-2022, demonstrated that COVID-19 vaccine messaging platforms implemented in EDs resulted in a 12% increase in vaccine acceptance and an 8% increase in 30-day uptake (number needed to treat [NNT], 13).^7^ The second study (PROFLUVAXED), conducted in 2022-2023, showed a 26% increase in 30-day influenza vaccine uptake with intervention M (NNT, 4) and a 17% increase with intervention Q compared with control (NNT, 6).^8^ These previous findings underscore the potential of ED-based messaging interventions to enhance vaccine uptake among medically vulnerable populations.

Although vaccine uptake did not increase with messaging in the whole study population in this trial, the increase in uptake among participants in EDs that had access to the updated COVID-19 vaccines suggests that vaccine availability was an effect modifier of the messaging platforms, underscoring the importance of opportunity and convenience in vaccine uptake. This is consistent with the PROCOVAXED study finding that most participants received the COVID-19 vaccine in real time during their ED visit rather than later at other health care sites.^7^ Patients may be more willing to receive a vaccine in the ED immediately after receiving messaging. A narrow window of vaccine acceptance and opportunity convergence for underserved ED populations who lack primary care may be missed if vaccines are not readily available in EDs.

Beyond vaccine availability, our null findings may also reflect an underlying change in public sentiment regarding COVID-19 vaccines over time. A recent Pew research study found that 60% of those surveyed in October 2024 did not plan to get the updated vaccine, compared with 32% in September 2022.^19^ The percentage of individuals who believed they did not need the vaccine nearly doubled from September 2020 to October 2024.^19^ Other concerns about updated vaccines included fears about adverse effects, consistent with our group’s prior research on COVID-19 booster vaccine hesitancy in the ED.^6^ This public perception may be in part due to decreasing overall COVID-related hospitalizations and death since 2020.^20^ Nevertheless, the risk of death in patients hospitalized for COVID-19 infection remains greater than that of influenza,^21^ especially for older adults, infants, and people with underlying medical conditions.^20^ Vaccines are a mainstay for reducing not just mortality but also postacute sequelae of COVID-19.^22^

Our research illuminates how federal public health policy decisions can affect ground-level public health interventions. During the public health emergency, 1 ED was able to provide free COVID-19 vaccines to nearly 1000 highly underserved individuals.^23^ Without funding for vaccines, these real-time, opportunity-based vaccine delivery programs may no longer be feasible. Furthermore, the cost of vaccines may also place them out of reach for outpatient uninsured and underinsured populations. Given that updated COVID-19 vaccines have been shown to reduce the likelihood of COVID-19-associated hospitalization and ED or urgent care encounters,^24^ future work may include cost-effectiveness analyses to gauge the return on investment of federally funded vaccine allocation and administration programs in EDs.

### Limitations

The use of EHR review limited our primary outcome assessment; although these reviews included immunizations from multiple sites, participants may have received vaccines at sites not captured within the EHR. Given that 25% of participants did not consent to be called, even complete telephone follow-up would have missed patients.

The limited availability of vaccines, with some study sites not having the vaccine at all and others having to source it from their hospital’s central pharmacy, limited ED administration. Only about 1 in 10 participants who indicated they would accept an updated COVID-19 vaccine received one during their ED visit, either because it was unavailable or because there were delays in obtaining it from the central pharmacy. This suggests a need to stock vaccines in ED pharmacies to facilitate timely administration. Overall, these limitations may have biased our findings toward a null result.

Although we could not adjust for time or site in our analysis, the roughly equal distribution of characteristics across study arms suggests randomization was likely adequate to control for hidden confounders. All EDs were in urban cities; thus, our findings may be less generalizable to rural settings.

## Conclusions

While neither tailored vaccine messaging nor simple inquiry increased the 30-day uptake of the updated COVID-19 vaccine among ED patients, ED vaccine availability emerged as an important effect modifier. The findings suggest ensuring vaccines are available in ED pharmacies, coupled with culturally tailored messaging, holds promise for increasing vaccination rates and improving public health outcomes among underserved populations who do not otherwise have access to primary care.

## Notes

Supplement 1.
Trial Protocol

Supplement 2. **eTable 1.** Sample 15-Day Block Randomization Scheme **eMethods 1.** PROBOOSTVAXED Intake Survey **eMethods 2.** PROBOOSTVAXED Vaccine Acceptance Survey **eFigure.** Study Flow and Procedures **eTable 2.** Primary and Secondary Outcomes, Stratified by Vaccine Availability

Supplement 3.
Data Sharing Statement
