# Supplementary material for: COVID-19 Booster Vaccine Messaging in Emergency Departments: A Cluster Randomized Clinical Trial
Source: JAMA Netw Open. 2025 Oct 15;8(10):e2537655. doi: 10.1001/jamanetworkopen.2025.37655 (PMC12529187; doi:10.1001/jamanetworkopen.2025.37655)
Supplement: Supplement 1. — Trial Protocol [file jamanetwopen-e2537655-s001.pdf]

Project Title: PROmotion of COVID-19 BOOSTer VA(X)ccination in the Emergency Department – PROBOOSTVAXED

R01 AI166967-01

Sub-study: RTC for COVID-19 booster vaccine messaging platforms

Date of Document: October 28, 2023

Study Procedures Manual for PROBOOSTVAXED – A Cluster Randomized Trial:

Contents

I. Overview ... 3
II. IRB ... 5
III. Deposition of Protocol into ClinicalTrials.Gov ... 5
IV. Setting and Sites ... 5
V. Randomization ... 5
VI. Site Orientation and Training ... 6
VII. Study Hotline and Quality Assurance ... 6
VIII. Recruitment, Inclusions, Exclusions, and Consent ... 6
IX. Study Procedures: Intervention Blocks ... 7
X. Study Procedures: Control Blocks ... 10
XI. Research Staff Informing ED Providers When Participants Will Accept COVID-19 Booster Vaccine for Intervention M and Intervention Q Arms ... 11
XII. Consents and Rationale ... 12
XIII. Primary Outcomes and Ascertainment ... 12
XIV. Data Recording and Entry ... 14
XV. Data Analysis ... 14
XVI. Data Management Plan ... 16
XVII. Sample Size Considerations ... 17

I. Overview

On January 22, 2020, Acting Health and Human Services Secretary Norris Cochran declared COVID-19 a national public health emergency, an action that eventually enabled emergency authorization for free COVID-19 vaccines, testing and treatments. This public health declaration has been renewed seven times for 90-day intervals, as required under section 319 of the Public Health Service (PHS) Act. Under this declaration over the past two years, the US government has provided full support and distribution of COVID-19 vaccines and therapeutics (antibody therapies, remdesivir, and nirmatrelvir/ritonavir), such that they have been widely available and free of charge to all Americans. Although these measures have not, by any means, ended the pandemic, it is clear from multiple epidemiologic modeling studies that they have decreased hospitalizations and saved hundreds of thousands of lives. They have also led to a narrowing of the profound morbidity and mortality disparities gap that was seen in minority populations in the first two waves of the pandemic.

The PROCOVAXED trial was a multicenter study that sought to decrease COVID-19 vaccine hesitancy and increase COVID-19 vaccine uptake through the use of vaccine messaging platforms in the emergency department (ED). In this trial, we found that implementation of our COVID-19 messaging platforms (videos, information sheets and scripted, direct messaging) were associated with greater COVID-19 vaccine acceptance and uptake among unvaccinated ED patients (Rodriguez RM, Nichol G, Eucker SA, et al. Effect of COVID-19 Vaccine Messaging Platforms in Emergency Departments on Vaccine Acceptance and Uptake: A Cluster Randomized Clinical Trial. *JAMA Intern Med.* 2023;183(2):115–123. doi:10.1001/jamainternmed.2022.5909).

In January 2022, we extended PROCOVAXED by shifting the focus to ***vaccinated*** ED patients to examine the timely and critically important topic of booster vaccine hesitancy in underserved ED populations. To better characterize COVID-19 booster hesitancy, we performed a cross-sectional study at five high-volume, safety-net hospital EDs in four cities (San Francisco, Philadelphia, Seattle, and Durham, NC) using survey tools to gather quantitative data on vaccination status, demographic variables, usual source of care, and attitudes toward booster vaccination. Of 771 participants who had completed their full initial series, 316 (41%) had not received any booster vaccine. Among these 316 non-boosted participants, 179 (57%, 95% CI 51-62) stated they would decline or were unsure whether they would accept a booster vaccine if it was offered to them (i.e., booster-hesitant). We found the following associations with booster vaccine hesitancy: age 35-49 years vs age 18-34 years (OR 1.16, 95% CI 0.99-1.36); Asian vs White race (OR 0.21, 95% CI 0.05-0.93); Hispanic/Latino vs White ethnicity (OR 1.59, 95% CI 0.82-3.09); primary language non-English vs English (OR 2.35, 95% CI 1.49-3.71); and Republican vs Democrat party affiliation (OR 6.07, 95% CI 4.21-8.75). The three most common reasons for booster vaccine hesitancy were a preference to wait for more information (25%), concerns about side effects and safety (24%), and “I don’t need one because I’m fully vaccinated” (20%).

Recognizing the ED as a unique opportunity to address COVID-19 booster vaccine hesitancy in underserved populations, we will launch the PROmotion of COVID-19 BOOSTer VA(X)ccination in the Emergency Department (PROBOOSTVAXED) trial as an extension of the PROCOVAXED trial, seeking to increase COVID-19 *booster* vaccine acceptance and uptake among vaccinated ED patients. Because of Omicron variant-associated surges during the COVID-19 pandemic with corresponding research staff illness and ED overcrowding, we found wide week-to-week fluctuations in enrollment in the PROCOVAXED study. To reduce this variability of enrollment, we have changed the unit of randomization from 1-week to 1-day in the PROBOOSTVAXED trial.

IMPORTANT CHANGE: Under intense political pressure and in response to Congress' reluctance to spend more on COVID-19 pandemic relief, the Biden administration recently made two announcements: 1) the national public health emergency declaration regarding the COVID-19 pandemic will be left to expire as of January 11, 2023, and 2) the administration will end their provision of free COVID-19 vaccines, testing and therapeutics, shifting the costs for vaccines, testing and treatments to health insurers and patients via commercial payment mechanisms. Because of these changes in reimbursement, we have been informed that only some of the PROBOOSTVAXED study sites will have COVID-19 vaccines available for distribution in the ED, whereas others will not. Nevertheless, we will proceed with the same research protocol, however, we will be performing additional subgroup analyses that compare 30-day vaccine uptake in sites with available COVID-19 vaccine to those without for the intervention and control arms.

Specific Aim I: To determine whether implementation of COVID-19 booster vaccine trusted messaging platforms is associated with increased booster vaccine uptake in vaccinated ED patients. At five EDs (Zuckerberg San Francisco General, UCSF Parnassus Medical Center [San Francisco, CA], Thomas Jefferson University Hospital [Philadelphia, PA], Ben Taub Hospital [Houston, TX], Duke University Medical Center [Durham, NC]), we will conduct a cluster-randomized controlled trial of the implementation of PROBOOSTVAXED trusted messaging platforms, with 30-day booster vaccine uptake as the primary outcome and booster vaccine uptake in the ED as a secondary outcome. *Hypothesis: Implementation of PROBOOSTVAXED trusted messaging platforms in EDs will be associated with increased 30-day booster vaccine uptake in vaccinated ED patients.*

Specific Aim II: To determine whether implementation of COVID-19 booster vaccine trusted messaging platforms in EDs is associated with increased booster vaccine acceptance in vaccinated ED patients. For this specific aim, booster vaccine acceptance in the ED assessed via ED survey will be the primary outcome. *Hypothesis: Implementation of PROBOOSTVAXED trusted messaging platforms in EDs will be associated with increased booster vaccine acceptance in vaccinated ED patients.*

Specific Aim III: To determine whether implementation of a protocol in which ED patients are asked whether they will accept a COVID-19 booster vaccine in the ED is associated with increased booster vaccine uptake in vaccinated ED patients. *Hypothesis: Implementation of an ED protocol in which patients are asked whether they will accept a COVID-19 booster vaccine will be associated with increased 30-day booster vaccine uptake in vaccinated ED patients.*

General Design: This is a three-arm cluster-randomized controlled trial (CRCT) to accomplish Specific Aims I, II, and III.

Study arms

PROBOOSTVAXED Intervention M (Messaging + Vaccine Question)	Intervention Q (Vaccine Question, No Messaging)	Control (No Messaging, No Vaccine Question)
• Vaccine messaging given<br>• Vaccine acceptance	• No vaccine messaging<br>• Vaccine acceptance	• No vaccine messaging<br>• No vaccine acceptance

question asked	question asked	question

Primary Outcome for Specific Aims I and III: Booster Vaccine Uptake in the ED

The primary outcome for Specific Aims I and III is **30-Day COVID-19 Booster Vaccine Uptake**, which will be ascertained by review of ED electronic health records (EHRs) at 30 days and follow-up phone calls. For Specific Aim I, this primary outcome will be compared between the Intervention M arm (Messaging + Vaccine Acceptance Question) and the Control arm (No Messaging, No Vaccine Acceptance Question). For Specific Aim III, this primary outcome will be compared between the Intervention Q arm (Vaccine Acceptance Question, No Messaging) and the Control arm (No Messaging, No Vaccine Acceptance Question).

Primary Outcome for Specific Aim II: Booster Vaccine Acceptance in the ED

The primary outcome of Specific Aim II is **COVID-19 Booster Vaccine Acceptance** in the ED, which will be ascertained by a survey question of study participants in the ED. This outcome will be compared between the Intervention M arm (Messaging + Vaccine Acceptance Question) and the Intervention Q arm (Vaccine Acceptance Question, No Messaging).

II. IRB

We will submit our protocol to the UCSF Committee on Human Research as a modification. We will continue with multi-site reliance mechanism for the PROCOVAXED study as per NIH guidelines for randomized trials.

III. Deposition of Protocol into ClinicalTrials.Gov

As per federal regulations, we will deposit our full study protocol into the repository https://clinicaltrials.gov/.

IV. Setting and Sites

We will conduct this over six months (mid-September 2023 to February 28, 2024) at five high-volume EDs in four cities: (Zuckerberg San Francisco General, UCSF Parnassus Medical Center [San Francisco, CA], Thomas Jefferson University Hospital [Philadelphia, PA], Ben Taub Hospital [Houston, TX], Duke University Medical Center [Durham, NC]). We have chosen this time-period to coincide with typical waves of the COVID-19 pandemic.

V. Randomization

Sites will be assigned be assigned to a condition for a day. Randomization within each of the site uses pseudorandom number to permute blocks of time. The blocks consist of 15 days duration during which each condition appears for 5 days. Hence, in any 15 day period there will be a balance of interventions within each of the sites. The particular days of each week (Monday, Tuesday, Wednesday, Thursday and Friday) for each of the study arms will thus vary randomly. This randomization scheme will minimize secular trends (changes in perceptions about the booster vaccine that may occur through the course of various pandemic waves). We will generate a full study calendar based on this randomization scheme. To try to maintain masking of allocation, sites will be given a blacked-out study calendar and will be instructed to

open the calendar for a particular study day the morning of that study day (other than that study day, the rest of the calendar will remain blacked out).

VI. Site Orientation and Training

The Core UCSF Site will develop orientation materials to familiarize the ED Sites with the study protocol. Each site will employ one or more Clinical Research Coordinators (CRCs), who will report to the Site PI and be responsible for day-to-day study implementation. We will develop and disseminate a manual of operating procedures (MOP) with standard personnel training methods, including education kits with scripts, summary cards, and PowerPoint presentations to assist coordinators in the orientation of site clinicians and other staff to our study protocol. We will convene ZOOM conference calls to review this summary and develop plans for optimization of PROBOOSTVAXED messaging platforms to improve usability and workflow. We will refine procedures with updates delivered to the site PIs during weekly ZOOM conferences.

VII. Study Hotline and Quality Assurance

We will maintain a study hotline and encourage study personnel to contact the PI and Central Study Coordinator for all issues and queries. Hotline hours will be during primary study hours (weekday 8 a.m. to 5 p.m. PST).

We will enact rigorous methods for clinical trial quality assurance and performance improvement, including: 1) systematic review of enrollment logs, 2) weekly audits of random samples of data for accuracy and missing elements, and 3) structured review of protocol deviations or violations. The Central Study Coordinator will prepare monthly summary report cards, tabulating individual site quality assurance metrics for review during scheduled Steering Committee calls. The overall study PI (Dr. Rodriguez) will discuss site-specific data with site PIs individually and summarize these data collectively during Steering Committee calls, with prompt dissemination of plans for process improvement.

VIII. Recruitment, Inclusions, Exclusions, and Consent

Practical budget considerations and limits on research personnel in patient care areas during the COVID-19 pandemic, preclude 24/7 delivery of the PROBOOSTVAXED trusted messaging platforms and enrollment in this study. We will use a convenience sample technique to approach all eligible adult patients who present to our study EDs during 8-hour (one-day) blocks, typically beginning at approximately 9 a.m. and continuing to approximately 5 pm. Sites will be given leeway to vary their particular study time hours, as long as these study hours remain consistent from week to week.

Inclusions will be:

1) Age > 18 years
2) Presenting to ED
3) No receipt of COVID-19 booster within prior 6 months
4) Able to provide informed consent
5) Fluent in English or Spanish
6) Anticipated ability to complete study intervention in ED i.e., able to watch a 3-minute videoclip

Reviewing ED triage information, we will exclude patients with the following characteristics:

1) Major trauma such that it will preclude survey
2) Inability to participate in a survey because of intoxication, altered mental status, or critical illness
3) Incarceration
4) Psychiatric hold
5) Patients who state that they have already received a bivalent COVID-19 booster vaccine or other COVID-19 vaccine within the prior 6 months
6) Patients who are in the ED for suspected acute COVID-19

NOTE: For all three study arms, study procedures should be performed in patient waiting times and not interfere or disrupt patient care in any way.

IX. Study Procedures: Intervention Blocks

<u>Procedures and workflow during PROBOOSTVAXED Intervention M Study Arm</u>

The anticipated flow of the study during the Intervention M study blocks is summarized in Figure 1. CRCs and research personnel will begin by setting up their home base of consents and platforms (video clips, printed materials, and scripts for messaging).

Figure 1: Intervention M Arm Study Flow

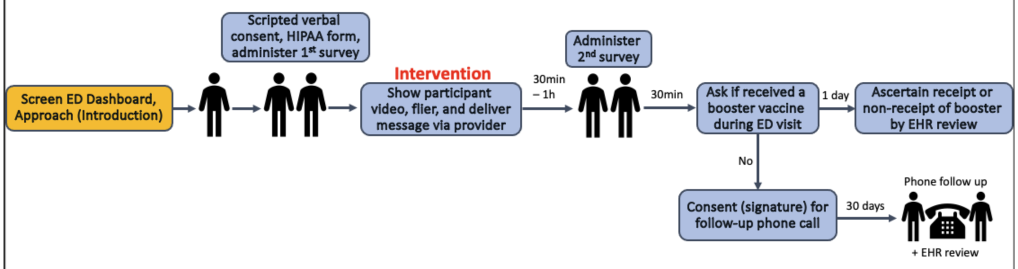

Introduction to ED Staff: Clinical Research Coordinators (CRCs) will set up their workstation in the ED and introduce themselves to ED staff (nurses, physicians, and mid-levels), informing them that they will be doing the PROBOOSTVAXED study that day. **They will avoid telling providers whether this is an intervention versus control arm.**

Initial Screening and Scripted Consent for Surveys: CRCs will review ED dashboards for inclusion and exclusion information. When an eligible patient is identified, the CRC will ask the nurse or doctor caring for the patient whether it is okay for them to approach the patient about the study. For provider approved patients: CRCs will approach eligible patients and deliver a scripted consent for two short surveys: the (Pre-intervention) Intake Survey and the (Post-Intervention) Vaccine Acceptance Survey. See Scripted Consent for the Intervention M period. **They will also get written HIPAA authorization for review of their ED EHR. If the patient does not agree to this HIPAA review of their EHR, they will be excluded from the study.** Participants will not be compensated for participation.

CRCs will complete screening and enrollment log indicating whether they agreed to participate. If they agreed to participate, the CRC will assign a Study ID#.

Intake Survey: We will administer the INTAKE SURVEY to participants. CRCs will have the option of inputting surveys to REDCap on iPads in real time or using paper surveys (and later inputting into REDCap). These surveys are to be delivered orally (CRC asks questions), not via handing them out. The Intake Surveys are the same for all three arms of the study.

Intervention M (messaging): The intervention will consist of three messaging platforms that were developed specifically to reduce booster vaccine hesitancy. All platforms have been reviewed by the UCSF Committee on Human Research.

1) Video clips – short (approximately 3-minute) Public Service Announcement type videos to be viewed by participant using a QR code on their smartphone. If no smartphone is available, the video will be shown to the participant on an iPad.
2) Printed materials – one page information sheets handed to subjects by CRCs.
3) Face to face messaging – short (< 1 minute), scripted message from the patient's providers in the ED (nurse or provider)

Each site will maintain a library of
A. 5 versions of the videos – the version used in any participant will be tailored to that participant's stated race/ethnicity. See *** below
B. 5 versions of printed flyers – likewise, the version will be tailored to the participant's stated race/ethnicity. See *** below
C. 1 version of scripted message to be delivered in English or Spanish.

COVID-19 Booster Vaccine Flyer, Videos, and Telling Provider to Deliver Message: Interventions will be delivered in real-time patient visits in site EDs, during waiting times such that they will not interfere with patient care. At the end of the survey, the CRC will deliver the booster vaccine information flyer and ask the patient if they will watch a short video about booster vaccines. If they agree to watch the video, the CRC will give them a QR code to view the video on their smartphone. If they do not have a smartphone, they will show them video on an iPad. After finishing with the video, the CRC will tell the subject that they will be back in about an hour for the Vaccine Acceptance survey. The CRC will then leave the room and ask the patient's primary provider (doctor, mid-level practitioner, or nurse) to deliver the booster vaccine message (hand them the scripted message). This message is short and should not significantly impact provider workflow. Notably, vaccine messaging is recommended in the ED by the American College of Emergency Physicians and other health care organizations (Centers for Disease Control).

***We will deliver messaging from our platform libraries in patients' preferred language (English, Spanish). To the extent possible, we will follow recommendations to choose platforms from site libraries that match video clip and printed material messengers with subjects' likely preferences for race, ethnicity, age, and gender (e.g., Latinx messenger on video clip with Latinx participant).

Vaccine Acceptance Survey (Post-Intervention) in the ED: We will administer the Vaccine Acceptance Survey: INTERVENTION GROUP at some time (generally 30 minutes but up to 3 hours) after the Intake Survey.

Primary and Other Outcome Ascertainment: Primary outcome ascertainment of 30-day uptake of booster vaccine will occur in three ways:

1) Ascertainment of vaccination in the ED by direct questioning and review of EHR records.
2) Blinded review of EHR at 30-days
3) For participants who have not received a booster vaccine upon questioning in the ED, staff will ask whether they will agree to phone follow-up at 30 days. For those agreeing to follow-up, we will obtain written consent for phone follow-up (phone follow-up consent form). We will then ask participants for their best phone number(s) to reach them for a follow-up phone call. Sites will maintain a separate password protected database of subject IDs and follow-up phone numbers.

This method will assure that all study participants, even those who refuse phone follow-up, will have at least two ways of study outcome ascertainment (1 and 2 above).

Secondary outcome ascertainment of booster vaccination in the ED will occur in two ways:

1) Direct questioning of participants and their providers in the ED: Research staff will ask participants and their ED providers whether the participant received a booster vaccine in the ED one hour but up to 6-hours after the Vaccine Acceptance Survey. Notably, ED patient visits are variable such that not all patients have stays lasting greater than an hour and a half. Research staff should endeavor to complete this ascertainment prior to discharge, even if that ascertainment occurs before an hour after the Vaccine Acceptance survey.
2) Review of each participant's ED EHR by research staff on the next workday after their index ED visit to confirm receipt (or non-receipt) of a booster vaccine in the ED. **This EHR review will be conducted in a blinded fashion – the research staff person reviewing the EHR will be unaware of participant's study group assignment.**

<u>Procedures and workflow during PROBOOSTVAXED Intervention Q Study Arm</u>

The workflow during this arm is identical to the Intervention M Arm except there will be no messaging platforms delivered. The anticipated flow of the study during **Intervention Q Blocks** is summarized in Figure 2.

Figure 2: Intervention Q Arm Study Flow

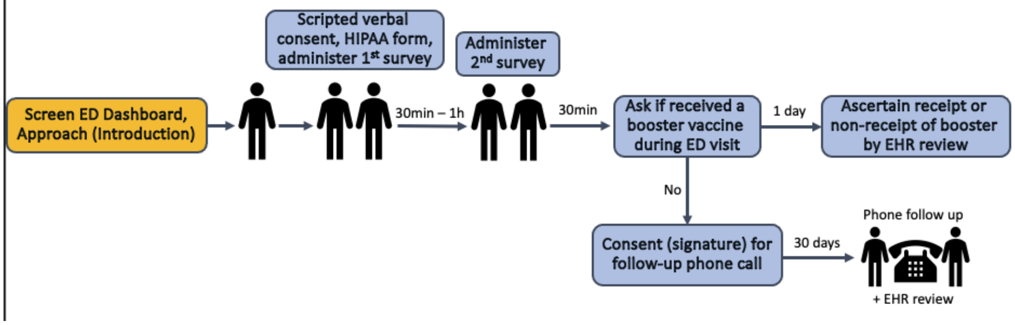

Introduction to ED Staff: Clinical Research Coordinators (CRCs) will set up their workstation in the ED and introduce themselves to ED staff (nurses, physicians, and mid-levels), informing them that they will be doing the PROBOOSTVAXED study that day. **They will avoid telling providers whether this is an intervention versus control arm.**

Initial Screening and Scripted Consent for Surveys: CRCs will review ED dashboards for inclusion and exclusion information. When an eligible patient is identified, the CRC will ask the nurse or doctor caring for the patient whether it is okay for them to approach the patient about the study. For provider approved patients: CRCs will approach eligible patients and deliver a scripted consent for two short surveys: the (Pre-intervention) Intake Survey and the (Post-Intervention) Vaccine Acceptance Survey. See Scripted Consent for the No Messaging arm period.

CRCs will complete screening and enrollment log indicating whether they agreed to participate. If they agreed to participate, the CRC will assign a Study ID#.

Intake Survey: We will administer the INTAKE SURVEY to participants. CRCs will have the option of inputting surveys to REDCap on iPads in real time or using paper surveys (and later inputting into REDCap). These surveys are to be delivered orally (CRC asks questions), not via handing them out. The Intake Surveys are the same for all three arms of the study.

Vaccine Acceptance Survey: We will administer the Vaccine Acceptance Survey at some time (generally 30 minutes but up to 3 hours) after the Intake Survey. The surveys in the control group retain the same key primary and secondary outcome questions as in the intervention group Vaccine Acceptance surveys. See Vaccine Acceptance Survey: No Messaging arm.

Primary and Other Outcome Ascertainment: Ascertainment of primary and secondary outcomes will occur in the same manner as in the Intervention M arm.

X. Study Procedures: Control Blocks

The workflow during this arm is identical to the Intervention Q arm except there will be no Vaccine Acceptance Question survey. The anticipated flow of the study during control blocks is summarized in Figure 3.

Figure 3: Control Arm Study Flow

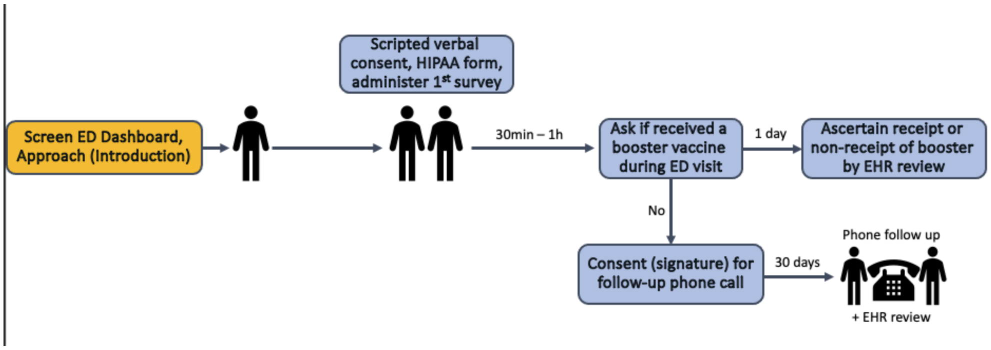

Introduction to ED Staff: Clinical Research Coordinators (CRCs) will set up their workstation in the ED and introduce themselves to ED staff (nurses, physicians, and mid-levels), informing them that they will be doing the PROBOOSTVAXED study that day. **They will avoid telling providers whether this is an intervention versus control arm.**

Initial Screening and Scripted Consent for Surveys: CRCs will review ED dashboards for inclusion and exclusion information. When an eligible patient is identified, the CRC will ask the nurse or doctor caring for the patient whether it is okay for them to approach the patient about the study. For provider approved patients: CRCs will approach eligible patients and deliver a scripted consent for two short surveys – the Intake Survey and the (Post-Intervention) Vaccine Acceptance Survey. See Scripted Consent for the No Messaging arm period.

CRCs will complete screening and enrollment log indicating whether or not they agreed to participate. If they agreed to participate, the CRC will assign a Study ID#.

Intake Survey: We will administer the INTAKE SURVEY to participants. CRCs will have the option of inputting surveys to REDCap on iPads in real time or using paper surveys (and later inputting into REDCap). These surveys are to be delivered orally (CRC asks questions), not via handing them out. The Intake Surveys are the same for all three arms of the study.

Primary and Other Outcome Ascertainment: Ascertainment of primary and secondary outcomes will occur in the same manner as in the intervention arms.

XI. Research Staff Informing ED Providers When Participants Will Accept COVID-19 Booster Vaccine for Intervention M and Intervention Q Arms

Only some study site EDs will have the capability of administering COVID-19 booster vaccines during the initial ED visit. The last question in the Vaccine Acceptance Survey **in both the Intervention M and Intervention Q arms** of the study is "*Would you accept the COVID-19 booster vaccine in the emergency department today if your doctor asked you?*" For study site EDs capable of administering the COVID-19 vaccine, when a participant says they will accept the vaccine, the CRC or research staff will ask the participant if it is okay to notify that participant's ED provider(s) – nurse and/or physician that they said they will accept the vaccine

and confirm whether or not they receive it in the ED. Research staff will not tell patients that they qualify for the booster vaccine and will not advise them in any manner. They will, however, inform the patient that if they choose to accept the vaccine, they may be liable for the vaccine's cost, depending on their medical insurance coverage. If participants agree to notification of the ED provider, research staff will notify the ED provider that they stated they will accept the vaccine. They will not tell the provider that they meet criteria for the booster vaccine and will not push that they get vaccinated.

XII. Consents and Rationale

We will obtain scripted verbal consent for Intake surveys in the same manner that we have conducted with the PROCOVAXED study, which is nearly identical in design and scope. We will obtain written HIPAA consent for EHR review and separate written consent for 30-day follow-up phone calls.

With regards to consent for delivery of the messaging intervention, we must emphasize that messaging for vaccine hesitancy is firmly a part of standard best-practice emergency department care (messaging of this type is currently be enacted in EDs across the US). Delivery of the vaccine messaging platforms is therefore an accepted common best practice not requiring consent. To add an extra layer of consent could lead to even greater vaccine hesitancy. We therefore plan the following processes with verbal assent for the Intervention:

1) At the end of the Intake Survey, asking patients if they are willing to watch a booster vaccine messaging video(s). If the patient says *Yes*, then we will play the video. If the patient says *No*, we will not play the video.
2) Asking participants whether they are willing to read an informational flyer about booster vaccines. If the patient says *Yes*, then we will give them the flyer. If the patient says *No*, we will not give them the flyer.
3) Handing the participant's ED provider(s) the scripted message about booster vaccines to deliver to the participant. Research staff will not mandate or check with providers whether they deliver the message.

XIII. Primary Outcomes and Ascertainment

Primary Outcome and Ascertainment of Specific Aim I

Our primary outcome for Specific Aim I is 30-day booster vaccine uptake, **comparing the Intervention M arm versus the Control (No Messaging, No Vaccine Acceptance Question) arm**.

Primary and Other Outcome Ascertainment: Primary outcome for Specific Aim I is booster vaccine uptake (at any vaccination location) within 30 days after participants' index ED visit, comparing the Intervention M arm versus the Control arm. For ascertainment of this outcome, we will:

1) Review the EHR the day after participants' index visit (as described for the secondary outcome ascertainment below).
2) Review the EHR at 30 days for receipt of a booster vaccine.

3) Conduct follow-up phone calls (*Have you received a booster vaccine since your emergency department visit?)* 30 days after index ED visits for those who did not get the vaccine in the ED and who consented to follow up.

Secondary outcome (vaccination in the ED) ascertainment will occur by review of each participant's ED EHR by research staff on the next workday after their index ED visit. Staff will check the EHR to confirm receipt (or non-receipt) of a booster vaccine in the ED. This review will be conducted in a blinded fashion – the research staff person reviewing the EHR will be unaware of participant's study group assignment.

Participants who have confirmed receipt by EHR review will be deemed "vaccinated in the ED". Conversely, participants who do not have confirmed receipt by review will be deemed to be "not vaccinated in the ED".

Outcome and Ascertainment of Specific Aim II

Our outcome for Specific Aim II is booster vaccine acceptance (defined as a response of "*yes*" to the question, "*Would you accept the booster vaccine in the emergency department today if your doctor asked you?*"), comparing the Intervention M arm versus Intervention Q arm. This outcome will be ascertained during the Vaccine Acceptance Survey in both arms.

Outcomes and Ascertainment of Specific Aim III

Our outcome for Specific Aim III is 30-day booster vaccine uptake (at any vaccination location), **comparing the** Intervention Q arm (Vaccine Acceptance Question, No Messaging) versus the Control arm (No Messaging, No Vaccine Acceptance Question). This outcome will be ascertained in the same manner as the primary outcome of Specific Aim I.

A secondary outcome for Specific Aim III is booster vaccination in the ED, comparing the Intervention Q arm versus the Control arm. This outcome will be ascertained in the same manner as the secondary outcome of Specific Aim I.

30-Day Phone and EHR Follow Up

CRCs will only review EHR and conduct phone follow-up with study subjects who have given written consent for these follow-up techniques. CRCs will check the EHR at two time periods – the day after their index visit and, if not vaccinated during their ED visit, again 30 days after their visit. CRCs will review Master Data Flow daily (workdays) to determine which subjects have reached the 1-month follow-up period. By convention, we will use the next month's day that has the same number as the index study visit date, i.e., if the study index visit was on November 5, then the 1-month follow-up should occur on December 5. If December 5 falls on a weekend, then the CRC will use the next study workday (typically the next Monday) as the follow-up date. Study subject's medical record #s and telephone #s will be accessed from the Booster Vaccine Follow Up sheet. The CRC who conducts EHR and phone follow-up will be blinded to the subject's study group assignment (intervention vs control arms), i.e., a separate CRC who did not recruit at that site during that day will conduct this phone follow up.

1) The CRC will first review the EHR to determine whether there is any record of a booster vaccine received in the preceding time period from the study index visit. If there is a record

of vaccination, the CRC will record what date and where the participant received it (if available). See Follow-Up Data Collection form.

2) If there is no record of vaccination in the EHR, the CRC will proceed with a phone call to the study subject. See Follow-Up Phone Call Collection form. CRCs will enter follow up data on both the Master Data Flow and Follow up spreadsheets via REDCap links.
   a. If the patient does not answer the phone that morning, the CRC will place two more calls to the study subject over the next 2 workdays. They will vary the time of these calls to improve response.
   b. If the patient does not answer the phone by the third call, the CRC will leave a message with the phone # of the study team. No more calls will be initiated by the study team after this third call.

XIV. Data Recording and Entry

CRCs will record survey responses and other data via two mechanisms:

1) Direct entry into the Booster Vaccine Study REDCap database in real time during surveys via secure links
2) Recording onto paper forms first. Then entry of survey information and data after each participant enrollment.

CRCs will keep a running log of all study flow and enrollment, recording the following data for all patients approached: study date, study arm, “Yes” and “No” agreeing to surveys, delivery or non-receipt of messaging platforms, agreeing to receipt of study vaccines, ED vaccine availability, receipt of vaccines in the ED, “Yes” and “No” agreeing to follow-up calls and EHR review. See Master Data Flow.

XV. Data Analysis

Analysis for Specific Aim I

The primary study comparison is uptake (receipt) of a booster vaccine at any vaccination location within 30 days of the index ED visit, comparing participants seen on Intervention M dates with those seen on Control (No Messaging, No Vaccine Acceptance Question) dates to test our study hypothesis: *Implementation of PROBOOSTVAXED trusted messaging platforms in EDs will be associated with increased booster vaccine uptake in vaccinated ED patients.* This outcome will be ascertained by either review of a participant’s EHR 30 days after their index ED visit or a follow-up phone call.

There are three secondary comparisons for Specific Aim I:
1) Comparison of 30-day booster vaccine uptake between the Intervention M and Intervention Q arms
2) Receipt of a booster vaccine during the index ED visit, comparing participants seen on Intervention M dates with those seen on Control dates - ascertained by check of the participant’s EHR on the day after their ED visit

Outcomes will be compared using mixed logistic regression with a fixed effect for randomization assignment, a normally distributed random effect to allow for clustering by enrolling center, and restricted cubic splines to allow for secular trends during the study period. The treatment effects

will be tested by the coefficient for the fixed effect of study arm along with 95% confidence intervals.

Analysis for Specific Aim II

For Specific Aim II, we will compare outcomes in participants seen on Intervention M arm dates with those seen on Intervention Q arm dates to test our study hypothesis: *Implementation of PROBOOSTVAXED trusted messaging platforms in EDs will be associated with increased booster vaccine acceptance in vaccinated ED patients.*

The outcome is booster vaccine acceptance (defined as a response of "*yes*" to the question "*Would you accept the booster vaccine in the emergency department today if your doctor asked you?*"). This outcome will be compared using mixed logistic regression with a fixed effect for randomization assignment, a normally distributed random effect to allow for clustering by enrolling center, and restricted cubic splines to allow for secular trends during the study period. The treatment effects will be tested by the coefficient for the fixed effect of study arm along with 95% confidence intervals.

Analysis for Specific Aim III

For Specific Aim III, the outcome is uptake (receipt) of a booster vaccine at any vaccination location within 30 days of the index ED visit. We will compare outcomes in participants seen on Intervention Q arm dates with those seen on Control dates to test our study hypothesis: *Implementation of an ED protocol in which patients are asked whether they will accept a booster vaccine (and notifying ED providers when they say they will accept it) will be associated with increased booster vaccine uptake in vaccinated ED patients.*

A secondary outcome for Specific Aim III is receipt of a booster vaccine during the index ED visit, comparing participants seen on Intervention Q dates with those seen on Control dates - ascertained by check of the participant's EHR on the day after their ED visit.

Subgroup Analyses

Another focus of this research is on ED patients who lack primary care group, defined on the Intake survey question: "*Do you have a regular clinic or doctor for medical care?"* We will analyze outcomes according to the binary indicator of having primary care – *yes* versus *no* (and *unsure*).

We will additionally stratify outcomes by study site (representing different regions of the country and different communities), vaccine administration capability, age, gender, primary language, and race/ethnicity.

Subgroups will be tested by adding a subgroup by intervention interaction to the mixed logistic regression. A subgroup will be considered significant if the pairwise intervention by subgroup omnibus test is significant at the 0.05 level.

Rationale for time (1-day unit) cluster and consideration of Alternative Study Designs:

In the study Overview, we described our rationale for switching from a one-week unit to a one-day unit cluster. Our primary goal with this research is to determine whether real-world implementation of booster vaccine messaging as an ED-site level intervention results in greater acceptance and uptake of booster vaccines in vulnerable ED populations. Each site sees approximately 150-250 patients per day and applying or not applying the intervention (delivery of booster vaccine messaging) *for individual patients* in this high workflow, rapid patient turnover ED environment is simply impractical. Given that booster vaccine messaging may be seen and received by all patients non-selectively in the EDs, patient level randomization would result in high risk of cross-contamination between intervention and control arms. Therefore, removal of the messaging intervention from the site completely during specified time periods (1-day units) of Intervention Q, and removal of both interventions (messaging and the vaccine acceptance question) during Control days is the optimal approach. Although single switches of turning on the interventions at each site (i.e., stepped-wedge trial design) is easier to enact, changes in general population attitudes over time introduce bias that limit the validity of this trial method. We expect gradually increasing acceptance and uptake of the booster vaccine over time, which would introduce substantial bias toward the intervention. Finally, cost considerations and feasibility limit the number of sites in this trial, negating the potential advantages of a cross-over trial with randomization by sites. These practical and methodological benefits of the 1-day unit cluster RCT far outweigh the smaller sample size and easier analysis with an individual patient unit RCT or a stepped-wedge design.

Statistical approach: In terms of statistical approach, this is a superiority trial in which we seek to verify our central study hypothesis that provision of booster vaccine messaging will result in greater acceptance and uptake of the booster vaccine. Following the recommendations of Hussey and Hughes, our statistical analyses will focus on comparing the vaccine uptake rates during the time periods when booster vaccine messaging is in place (Intervention M) and when the system is not in place (Control - usual care) using mixed effects logistic models. The outcome of interest is the binary indicators of whether they have received a booster vaccine in the ED (uptake - yes/no). Models will include a normally distributed random center effect (on the logit scale) to accommodate potential within-center characteristics (e.g., case mix, demographics), as well as terms for time and intervention. Hypotheses testing will focus on the statistical significance of the intervention indicator. We will fit the mixed effects models using maximum likelihood and routines in Stata.

We will test our primary hypotheses and analyze outcomes according to the study arm (index visit in Intervention M day versus control day) to which patients were allocated, regardless of whether they received booster vaccine messaging or not - ***intention to treat analysis***.

In addition to the effects on *total* vaccine acceptance, we will also examine the effect of booster vaccine messaging on acceptance in patient sub-groups, especially African American and Latinx persons. Booster vaccine messaging may work for one patient sub-group and not others -- these additional analyses will guide future directions and modifications of booster vaccine messaging.

XVI. Data Management Plan

We will manage data using REDCap, hosted by the core site (UCSF) for secure data entry and management. Patient identifiers (medical record numbers and phone numbers) only link will be

to unique study ID numbers, which will be housed in files that are kept separate from other study data. We will develop a detailed data dictionary to ensure consistent standards across sites. We will reduce missing or erroneous data using the REDcap data quality tool.

XVII. Sample Size Considerations

The sample size calculations for this research are governed by hypothesis testing of Specific Aim I -- *Implementation of booster messaging platforms will be associated with increased booster vaccine uptake (primary outcome) in unvaccinated ED patients.* Considering the high benefit of increasing vaccine uptake and the negligible risk of the intervention (a trusted messaging program), even a small effect size of increased uptake would be a clinically important difference. By investigator consensus, we have determined that the intervention would be clinically useful if it increased booster vaccine uptake by 7%.

We base the sample size calculation on the comparison of the proportion of patients who accept the vaccine between the Intervention M and Control time periods using standard formulae for individual randomization. We have verified that these sample sizes are conservative by simulation of data using a mixed random effects model. Our baseline level of vaccine uptake in the control arm is estimated to be approximately 5%. With this uptake level we find that at an alpha=0.05 level and a power of at least 0.80, we will need to enroll 744 participants (248 in each arm) in the study to detect the difference of interest (a setting in which the vaccine uptake rate will increase by 7% during Intervention periods vs Control periods).
