# Supplementary material for: COVID-19 Booster Vaccine Messaging in Emergency Departments: A Cluster Randomized Clinical Trial
Source: JAMA Netw Open. 2025 Oct 15;8(10):e2537655. doi: 10.1001/jamanetworkopen.2025.37655 (PMC12529187; doi:10.1001/jamanetworkopen.2025.37655)
Supplement: Supplement 2. — eTable 1. Sample 15-Day Block Randomization Scheme eMethods 1. PROBOOSTVAXED Intake Survey eMethods 2. PROBOOSTVAXED Vaccine Acceptance Survey eFigure. Study Flow and Procedures eTable 2. Primary and Secondary Outcomes, Stratified by Vaccine Availability [file jamanetwopen-e2537655-s002.pdf]

Supplementary Online Content

Molina MF, Eucker SA, Rising KL, et al. COVID-19 booster vaccine messaging in emergency departments: a cluster randomized clinical trial. *JAMA Netw Open*. 2025;8(10):e2537655. doi:10.1001/jamanetworkopen.2025.37655

eTable 1. Sample 15-Day Block Randomization Scheme
eMethods 1. PROBOOSTVAXED Intake Survey
eMethods 2. PROBOOSTVAXED Vaccine Acceptance Survey
eFigure. Study Flow and Procedures
eTable 2. Primary and Secondary Outcomes, Stratified by Vaccine Availability

This supplementary material has been provided by the authors to give readers additional information about their work.

© 2025 Molina MF et al. *JAMA Network Open*.

Date	Site A	Site B	Site C	Site D	Site E	Site F
January 1, 2024	Intervention M	Control	Intervention M	Intervention Q	Control	Intervention Q
January 2, 2024	Intervention M	Control	Control	Intervention Q	Intervention Q	Intervention M
January 3, 2024	Control	Intervention Q	Intervention Q	Intervention M	Intervention M	Control
January 4, 2024	Intervention M	Control	Control	Intervention Q	Intervention Q	Intervention M
January 5, 2024	Control	Intervention Q	Intervention M	Intervention M	Control	Intervention Q
January 8, 2024	Intervention Q	Intervention M	Intervention Q	Control	Intervention M	Control
January 9, 2024	Intervention M	Control	Control	Intervention Q	Intervention Q	Intervention M
January 10, 2024	Control	Intervention Q	Intervention Q	Intervention M	Intervention M	Control
January 11, 2024	Intervention Q	Intervention M	Intervention Q	Control	Intervention M	Control
January 12, 2024	Intervention Q	Intervention M	Intervention M	Control	Control	Intervention Q
January 15, 2024	Intervention Q	Intervention M	Intervention M	Control	Control	Intervention Q
January 16, 2024	Intervention M	Control	Control	Intervention Q	Intervention Q	Intervention M
January 17, 2024	Intervention Q	Intervention M	Control	Control	Intervention Q	Intervention M
January 18, 2024	Control	Intervention Q	Intervention Q	Intervention M	Intervention M	Control

eTable 1: Sample 15-Day Block Randomization Scheme

© 2025 Molina MF et al. *JAMA Network Open*.

eMethods 1. PROBOOSTVAXED Intake Survey

PROBOOSTVAXED FIRST SURVEY (SAME FOR ALL STUDY ARMS)

Please complete

the survey below.

Thank you!

Study ID: [study_id_screening]	______________________________	
Date	______________________________	
Time	______________________________	
CRC Initials	______________________________	
Have you previously been diagnosed with COVID-19 or<br>19 test? / ¿Usted ha sido<br>diagnosticado con COVID-19 o ha tenido una prueba<br><br>ure / No estoy seguro(a) positiva de COVID-19 ?	◯<br>◯<br>◯	Yes / Sí had a positive COVID-<br>No / No<br><br>Uns
If yes:<br>How many times did you have COVID-19? / ¿Cuántas veces tuviste COVID-19?	◯<br>◯<br>◯<br>◯<br>◯<br>◯<br>◯	1<br>2<br>3<br>4<br>5<br>More than 5 / Más de 5<br>Unsure / No estoy seguro(a)
If yes:<br>6 meses	◯<br>◯<br>◯	1 to 6 months ago / Hace 1 a<br><br>6 months to 1 year ago /

© 2025 Molina MF et al. *JAMA Network Open*.

Hace 6 meses a un año When was the LAST time you had COVID-19?/ ¿Cuándo > 1 year ago / Hace más de un año
fue la ultima vez que tuviste COVID-19?

Have you ever had a COVID-19 vaccine?/ ¿Alguna vez vacuna contra COVID-19?	◯<br>◯<br>◯	Yes / Sí has recibido una<br>No /No<br>Unsure/ No estoy seguro(a)
If yes:<br><br>How many COVID-19 vaccines have you had?/ ¿Cuántas vacunas contra COVID-19 has recibido?	◯<br>◯<br>◯<br>◯<br>◯<br>◯	1<br>2<br>3<br>4<br>5<br>More than 5 / Más de 5
If yes:<br>de un año<br><br>When did you have your LAST COVID-19 vaccine? /<br>dos años<br>¿Cuando fue la última vez que se vacunó contra<br><br>Unsure / No estoy seguro COVID-19?	◯<br>◯<br>◯<br>◯	< 1 year ago / Hace menos<br><br>1-2 years ago / 1-2 años<br>> 2 years ago / Hace más de

© 2025 Molina MF et al. *JAMA Network Open*.

If yes (Check all that apply):<br><br>Tell us ALL of the places that you got COVID-19 vaccines? / Si es asi, indícanos TODOS los lugares donde recibiste las vacunas contra COVID-19 (marca todos los que correspondan).	☐ My doctor's office or clinic / Mi consultorio médico o clínica<br>☐ A pharmacy / Una farmacia<br>☐ An ER - emergency department/ Una sala de emergencias (depertamento de emergencias)<br>☐ A mass vaccination place / Un lugar de vacunación masiva<br>☐ An urgent care clinic (not my doctor or clinic) / Un clínica de atención de urgencia (que no es mi consultario médico ni clínica)<br>☐ Other / Otro<br>☐ I don't remember / No me acuerdo
Specify other	________________________
What is your gender? / ¿Cuál es su género?	◯ Male / Hombre<br>◯ Female / Mujer<br>◯ Transgender male / Hombre transgénero<br>◯ Transgender female / Mujer transgénero<br>◯ Non-binary, gender non-conforming / No binario, no conforme al género<br>◯ Other, Not Listed / Otro, No elinstado<br>◯ Prefer not to answer / Prefiero no contestar
Specify other / Para otro explique	________________________
Are you of Hispanic or Latino Origin? / ¿Es usted de origen hispano o latino?	◯ Yes/Sí<br>◯ No/No<br>◯ Prefer not to answer / Prefiero no contestar
Which best describes your race?/ ¿Cuál describe mejor tu raza? (Check all that apply) [OMB/HHS Standards]	☐ Indigenous/American Indian/Alaska Native / Indígena/ Americano indígena/ Nativo de Alaska<br>☐ Asian / Asiático<br>☐ Black or African American / Negro o afroamericano<br>☐ Native Hawaiian/Other Pacific Islander / Nativo de Hawái / Otra isla del Pacifico<br>☐ White / Blanco<br>☐ Some other race / Otra raza
Specify other	________________________
If Asian (select as many as apply):<br><br>Please specify which sub-category / Especifica la subcategoría	☐ Chinese / Chino<br>☐ Filipino / Filipino<br>☐ Asian Indian / Indio asiático<br>☐ Vietnamese/ Vietnamita<br>☐ Korean / Coreano<br>☐ Japanese / Japonés<br>☐ Other Asian (e.g., Pakistani, Cambodian, Hmong) / Otro asiático (por ejemplo, pakistaní, camboyano, hmong)
If Native Hawaiian/Other Pacific Islander:<br><br>Please specify sub-category (select as many as apply)/ Especifica la subcategoría	☐ Native Hawaiian/ Nativo Hawaiano<br>☐ Samoan/ Samoano<br>☐ Chamorro / Chamorro<br>☐ Other Pacific Islander (e.g., Tongan, Fijian, Mashallese) / Otro isleño del Pacífico (por ejemplo tongano, fiyiano, mashallese)

© 2025 Molina MF et al. *JAMA Network Open*.

What is your living situation today?/ ¿Cuál es tu situación de vivienda hoy?	◯ I have a steady place to live (home, apartment or other) / Tengo un lugar estable para vivir (casa, apartamento u otro)<br>◯ I have a place to live today, but I am worried about losing it in the future/ Tengo un lugar para vivir hoy, pero me preocupa perderlo en el futuro<br>◯ I do not have a steady place to live (I am temporarily staying with others, in a hotel, in a shelter, living outside on the street, on a beach, in a car, abandoned building, bus or train station, or in a park) / No tengo un lugar estable para vivir (estoy temporalmente alojado con otras personas, en un hotel, en un refugio, viviendo afuera en la calle, en una playa, en un automóvil, edificio abandonado, estación de autobús o tren, o en un parque)
Do you have any kind of health care coverage, including health insurance, prepaid plans such as HMOs, or government plans such as Medicaid, Medicare, or Indian Health Service? / ¿Tienes algún tipo de cobertura de atención médica, incluyendo seguro de salud, planes prepagos como HMO o planes gubernamentales como Medicaid, Medicare o el Servicio de Salud Indígena?	☐ I do not have health insurance / No tengo seguro de salud<br>☐ Medicare (65+ and older) / Medicare (65 años o más)<br>☐ Medicaid/State Insurance (Medical) / Medicaid/ Seguro estatal (Medical)<br>☐ Obamacare (Affordable Care Act) / Obamacare (Ley de cuidado de Saludo a Bajo Precio)<br>☐ Military (VA) / Militar (VA)<br>☐ Private / Commercial / Employer-based / Self-insured / Privado/ Comercial / Basado en el empleador/ Autoasegurado<br>☐ Kaiser / Kaiser<br>☐ Indian Health Service / Servicio de Salud Indígena<br>☐ Healthy San Francisco / Healthy San Francisco<br>☐ Other insurance / Otro seguro<br>☐ Unsure / No estoy seguro(a)
Specify other	______________________________
Do you have a regular clinic or doctor for medical care? / ¿Tienes una clínica o médico regular para recibir atención médica?	◯ Yes / Sí<br>◯ No / No<br>◯ Unsure / No estoy seguro(a)
If NO to regular doctor or clinic:<br><br>When was the last time you saw any doctor or had health care in the U.S. (besides today)?/ ¿Cuándo fue la última vez que viste a algún médico o recibiste atención médica en los Estados Unidos (además de hoy)?	◯ < 6 months / Menos de 6 meses<br>◯ 6 months to a year / De 6 meses a 1 año<br>◯ 1 year to 5 years / De 1 a 5 años<br>◯ > 5 years / Más de 5 años<br>◯ I have never seen a doctor in the U.S. / Nunca he visto a un médico en los Estados Unidos
If NO to regular doctor or clinic:<br><br>Where do you usually go when you are sick or need medical advice?/ ¿A dónde sueles ir cuando estás enfermo o necesitas consejo médico?	◯ An emergency department / Departamento de emergencias<br>◯ A clinic / Clínica<br>◯ Urgent care center / centro de atención de urgencias<br>◯ Other / Otro<br>◯ I don't ever go anywhere / no voy a ningún lado<br>◯ I have never been sick / Nunca he estado enfermo<br>◯ Unsure / No estoy seguro(a)

© 2025 Molina MF et al. *JAMA Network Open*.

Specify other	__________________________________
What is your primary language?/ Cuál es tu idioma principal?<br>[First language you ever spoke and/or language you speak at home] / [Primer idioma que hablaste y/o idioma que hablas en casa]	☐ English / Inglés<br>☐ Spanish / Español<br>☐ Cantonese/Mandarin / Chino (Mandarín/ Cantonés)<br>☐ Tagalog / Tagalo<br>☐ Arabic / Árabe<br>☐ Bengali / Begalí<br>☐ Other / Otro
Specify other	__________________________________
Is this an Intervention M Arm?	◯ Yes<br>◯ No
Hand patient the flyer and video, see below:<br><br>Flyer A - Mixed Race<br>Flyer B - Asian<br>Flyer C - African American<br>Flyer D - White<br><br>Spanish Only Flyer A or Spanish Only Flyer D - 'White'<br>Spanish Only Flyer A or Spanish Only Flyer C - 'Non-White Race'<br><br>Video QR 1 - White<br>Video QR 2 - African American<br>Video QR 3 - Spanish Speaking Only<br>Video QR 4 - Latino/Hispanic English<br>Video QR 5 - Mixed Race	
Did the participant watch the video?	◯ Yes, given video<br>◯ Yes, given but not completed (interrupted or other)<br>◯ No, refused video
If yes, which modality?	◯ QR code using their smartphone<br>◯ iPad, had phone but not able to scan QR code<br>◯ iPad, had phone but not able to stream due to poor connectivity<br>◯ iPad, did not have a phone
IF YES, which video was given?	◯ Video QR 1 - White<br>◯ Video QR 2 - African American<br>◯ Video QR 3 - Spanish Speaking Only<br>◯ Video QR 4 - Latino/Hispanic English<br>◯ Video QR 5 - Mixed Race
Did the participant receive the flyer?	◯ Yes, given flyer<br>◯ No, refused flyer

© 2025 Molina MF et al. *JAMA Network Open*.

IF YES, which flyer was given?	◯ Flyer A - Mixed Race<br>◯ Flyer B - Asian<br>◯ Flyer C - African American<br>◯ Flyer D - White<br>◯ Spanish Only Flyer A or Spanish Only Flyer D - 'White'<br>◯ Spanish Only Flyer A or Spanish Only Flyer C - 'Non-White Race'
Told the provider to deliver message?	◯ Yes<br>◯ No
IF YES, who did you tell to deliver the message?	◯ Physician<br>◯ Mid-level (NP, PA)<br>◯ Nurse
IF NO, explain why	__________________________________

© 2025 Molina MF et al. *JAMA Network Open*.

eMethods 2. PROBOOSTVAXED Vaccine Acceptance Survey

PROBOOSTVAXED SECOND SURVEY (SAME FOR INTERVENTION M AND INTERVENTION Q)

Please complete the survey below.

Thank you!

Study ID: [study_id_screening]	______________________________
Date and Time	______________________________
CRC Initials	______________________________
OF NOTE: These options are NOT in the same order as before. Please ensure you are choosing the correct intervention arm option<br><br>Study Arm: [study_arm_followup]	◯ Control<br>◯ Intervention Q<br>◯ Intervention M
Did the video(s) affect the way you feel about getting a COVID booster vaccine? / ¿Los videos afectaron la forma en que te sientes acerca de recibir la vacuna de refuerzo contra COVID?	◯ The video made it more likely that I will get a COVID booster vaccine / El video hizo que fuera más probable que reciba la vacuna de refuerzo contra COVID<br>◯ It did not affect the way I feel about getting a vaccine / No afectó la forma en que me siento acerca de recibir una vacuna<br>◯ It made it less likely that I will get a COVID booster vaccine / Hizo que fuera menos probable que reciba la vacuna de refuerzo contra COVID<br>◯ I did not watch the video / No vi el video
What would you change about the video(s)? / ¿Qué cambiarías acerca de estos videos?	◯ Nothing / Nada<br>◯ Make them shorter / Hacerlos más cortos<br>◯ Make them longer / Hacerlos más largos<br>◯ Use a different speaker on the video /Utiliza un presentador diferente en el video<br>◯ Other / otro
If you would use a different speaker, who would you use? / Si utilizarías a un presentador diferente, ¿a quién elegirías?	______________________________
Specify other	______________________________
Did the information sheet affect the way you feel about getting a COVID booster vaccine? / ¿La hoja de información afectó la forma en que te sientes acerca de recibir la vacuna de refuerzo contra COVID?	◯ The sheet made it more likely that I will get a COVID booster vaccine / La hoja hizo que fuera más probable que reciba la vacuna de refuerzo contra COVID<br>◯ It did not affect the way I feel about getting a vaccine / No afectó la forma en que me siento acerca de recibir una vacuna<br>◯ It made it less likely that I will get a COVID booster vaccine / Hizo que fuera menos probable que reciba la vacuna de refuerzo contra COVID<br>◯ I did not look at the information sheet / No vi la hoja de información

© 2025 Molina MF et al. *JAMA Network Open*.

What would you change about these printed materials? / ¿Qué cambiarías acerca de estos materiales impresos?	◯ Nothing / Nada<br>◯ Make them shorter/less information /Hacerlos más cortos/menos información<br>◯ Make them longer/provide more information / Hacerlos más largos/proveer más información<br>◯ Other / Otro
Specify other	____________________________
Did anyone (besides me) talk to you about COVID booster vaccines during your visit today? / ¿Alguien (además de mí) te habló sobre las vacunas de refuerzo contra COVID durante tu visita hoy?	◯ Yes/ Sí<br>◯ No / No<br>◯ Unsure / No estoy seguro
If yes:<br><br>Who was it? / ¿Quien fue?	◯ A doctor / Un doctor(a)<br>◯ A nurse / Un enfermero(a)<br>◯ Another provider / alguien más<br>◯ A social worker / Un(a) trabajador(a) social<br>◯ I don't know / No se
If yes:<br><br>Did that affect how you feel about getting the COVID booster vaccine? / ¿Eso afectó cómo te sientes acerca de recibir la vacuna de refuerzo contra COVID?	◯ The message made it more likely that I will get a COVID booster vaccine / El mensaje hizo que fuera más probable que reciba la vacuna de refuerzo contra COVID<br>◯ It did not affect the way I feel about getting a vaccine / No afectó la forma en que me siento acerca de recibir una vacuna<br>◯ It made it less likely that I will get a COVID booster vaccine / Hizo que fuera menos probable que reciba la vacuna de refuerzo contra COVID
If yes:<br><br>What would you change about these messages from the person? / ¿Qué cambiarías acerca de estos mensajes de la persona?	◯ Nothing / Nada<br>◯ Make them shorter/less information /Hacerlos más cortos/menos información<br>◯ Make them longer/provide more information / Hacerlos más largos/proveer más información<br>◯ Other / Otro
Specify other	____________________________
*ONLY ASK IF THEY RECEIVED MORE THAN 1 INTERVENTION<br><br>Which of the three (videos, information sheet or message from doctor) was the most useful to you? / De los tres (videos, hoja de información o mensaje del médico), ¿cuál fue el más útil para ti?	☐ Videos / Videos<br>☐ Print materials / Materiales Impresos<br>☐ Message from doctor / Mensaje del médico<br>☐ They were all about the same / Todos fueron igual de útiles<br>☐ Only received one intervention, not applicable / Solo recibí una intervención, no aplicable<br>☐ Other / Otro
Specify other	____________________________

© 2025 Molina MF et al. *JAMA Network Open*.

Would you accept the COVID booster vaccine in the emergency department today if your doctor or provider offered it to you? / Aceptarías la vacuna de refuerzo contra COVID en el departamento de emergencias hoy si tu médico o proveedor te la ofreciera?	◯ Yes / Sí<br>◯ No / No<br>◯ No, but I might consider getting it at some other time outside of the emergency department / No, pero podría considerar recibirla en algún otro momento fuera del departamento de emergencias.
IF NO or NO but I might consider getting at some other time to vaccine in the ED:<br><br>If you would not accept a booster vaccine, list the reason or reasons why not? / Si no aceptarías una vacuna de refuerzo, enumera la razón o razones por las cuales no lo harías.<br><br>(LET PATIENT ANSWER THE QUESTION AND CHOOSE BEST OPTION(S) BASED ON THEIR ANSWER; DO NOT READ ANSWER CHOICES)	☐ I need more information about a COVID booster / Necesito más información sobre una vacuna de refuerzo contra COVID<br>☐ I have concerns about side effects and safety of a booster vaccine / Tengo preocupaciones sobre los efectos secundarios y la seguridad de la vacuna de refuerzo<br>☐ I don't believe a COVID booster is needed because I'm already fully vaccinated / No creo que sea necesario recibir una vacuna de refuerzo porque ya estoy completamente vacunado(a)<br>☐ I have heard stories on media (online, social media, television, or radio) that give me doubts about BOOSTER vaccines / He escuchado historias en los medios de comunicación (en línea, redes sociales, televisión o radio) que me generan dudas sobre las vacunas de refuerzo<br>☐ I have concerns about cost of the booster / Tengo preocupaciones sobre el costo de la vacuna de refuerzo<br>☐ I'm tired of getting so many vaccines / Estoy cansado(a) de recibir tantas vacunas<br>☐ I'm not worried any more about Covid / Ya no me preocupa tanto COVID<br>☐ Other / Otro<br>☐ I will consider getting the Covid vaccine when I am not ill
Specify other	______________________________

If yes to vaccine in ED, READ the the following statement, then proceed with the next question:

If you receive a vaccine in the ED today, you maybe responsible for the cost, unless it is covered by your insurance.

Si recibes una vacuna en el departamento de emergencias hoy, es posible que seas responsable del costo, a menos que esté cubierto por tu seguro.

Would you STILL accept the COVID booster vaccine in the emergency department today if your doctor or provider offered it to you? / ¿AÚN aceptarías la vacuna de refuerzo contra COVID en el departamento de emergencias hoy si tu médico o proveedor te la ofreciera?	◯ Yes/ Sí<br>◯ No / No<br>◯ No, but I might consider getting it at some other time outside of the emergency department / No, pero podría considerar recibirla en algún otro momento fuera del departamento de emergencias

© 2025 Molina MF et al. *JAMA Network Open*.

IF NO or NO but I might consider getting at some other time to vaccine in the ED:<br><br>If you would not accept a booster vaccine, list the reason or reasons why not?/ Si no aceptarías una vacuna de refuerzo, enumera la razón o razones por las cuales no lo harías.<br><br>(LET PATIENT ANSWER THE QUESTION AND CHOOSE BEST OPTION(S) BASED ON THEIR ANSWER; DO NOT READ ANSWER CHOICES)	☐ I need more information about a COVID booster / Necesito más información sobre una vacuna de refuerzo contra COVID<br>☐ I have concerns about side effects and safety of a booster vaccine / Tengo preocupaciones sobre los efectos secundarios y la seguridad de la vacuna de refuerzo<br>☐ I don't believe a COVID booster is needed because I'm already fully vaccinated / No creo que sea necesario recibir una vacuna de refuerzo porque ya estoy completamente vacunado(a)<br>☐ I have heard stories on media (online, social media, television, or radio) that give me doubts about BOOSTER vaccines / He escuchado historias en los medios de comunicación (en línea, redes sociales, televisión o radio) que me generan dudas sobre las vacunas de refuerzo<br>☐ I have concerns about cost of the booster / Tengo preocupaciones sobre el costo de la vacuna de refuerzo<br>☐ I'm tired of getting so many vaccines / Estoy cansado(a) de recibir tantas vacunas<br>☐ I'm not worried any more about Covid / Ya no me preocupa tanto COVID<br>☐ Other / Otro<br>☐ I will consider getting the Covid vaccine when I am not ill
FOR CRC ONLY:<br><br>If yes, does your site have COVID vaccines available for ED patients?	◯ Yes<br>◯ No
If yes:<br><br>Can I notify your provider that you want the vaccine?/ ¿Puedo notificar a tu proveedor que quieres una vacuna?	◯ Yes/ Sí<br>◯ No / No<br>◯ No, but might consider getting it at some other time outside of the emergency room. No, pero podría considerar recibirla en algún otro momento fuera del departamento de emergencias.
If no, read the following statement:<br><br>Unfortunately, we do not have the COVID vaccine available in the emergency department today. Here is a list of places where you can get the vaccine. / Desafortunadamente, no tenemos la vacuna contra COVID disponible en el departamento de emergencias hoy. Aquí tienes una lista de lugares donde puedes obtener la vacuna.<br><br>FOR CRC ONLY:<br>HAND PARTICIPANT LIST OF RESOURCES. Were resources handed to the participant?	◯ Yes/ Sí<br>◯ No / No<br>◯ Patient declined / El paciente rachazó
Are you willing to have a research staff call you in a month and review your medical records?/ ¿Estás dispuesto/a a que un personal de investigación te llame en un mes y revise tus expedientes médicos?	◯ Yes (Proceed with written consent) / Sí<br>◯ No/ No

© 2025 Molina MF et al. *JAMA Network Open*.

If NO to notifying provider:<br><br>Can you provide a reason as to why?/ ¿Puedes proporcionar una razón por la cual?<br><br>(LET PATIENT ANSWER THE QUESTION AND CHOOSE BEST OPTION(S) BASED ON THEIR ANSWER; DO NOT READ ANSWER CHOICES)	◯ I need more information about a COVID booster / Necesito más información sobre una vacuna de refuerzo contra COVID<br>◯ I have concerns about side effects and safety of a booster vaccine / Tengo preocupaciones sobre los efectos secundarios y la seguridad de la vacuna de refuerzo<br>◯ I don't believe a COVID booster is needed because I'm already fully vaccinated / No creo que sea necesario recibir una vacuna de refuerzo porque ya estoy completamente vacunado(a)<br>◯ I have heard stories on media (online, social media, television, or radio) that give me doubts about BOOSTER vaccines / He escuchado historias en los medios de comunicación (en línea, redes sociales, televisión o radio) que me generan dudas sobre las vacunas de refuerzo<br>◯ I have concerns about cost of the booster / Tengo preocupaciones sobre el costo de la vacuna de refuerzo<br>◯ I'm tired of getting so many vaccines / Estoy cansado(a) de recibir tantas vacunas<br>◯ I'm not worried any more about Covid / Ya no me preocupa tanto COVID<br>◯ Other / Otro
Specify other	__________________________
If NO to vaccine in ED:<br>Are you willing to have a research staff call you in a month and review your medical records?/ Estás dispuesto(a) a que un personal de investigación te llame en un mes y revise tus expedientes médicos?	◯ Yes (Proceed with written consent)<br>◯ No
If the patient said yes to vaccine:<br><br>Are you willing to have research staff call them in a month and review their medical records? / ¿Estas dispuestos a que el personal de investigación le llame en un mes y revise sus expedientes médicos?	◯ Yes (Proceed with written consent)<br>◯ No
If CONTROL:<br>Are you willing to have a research staff call you in a month and review your medical records?/ Estás dispuesto(a) a que un personal de investigación te llame en un mes y revise tus expedientes médicos?	◯ Yes (Proceed with written consent)<br>◯ No
AT THE END OF ED VISIT ASK PATIENT OR PROVIDER:<br><br>Did patient receive a COVID-19 booster vaccine during their ED visit today? / ¿El paciente recibió ALGUNA vacuna durante su visita al departamento de emergencias hoy?	◯ Yes / Sí<br>◯ No / No<br>◯ Unsure/ No estoy seguro(a)
Which vaccine did they receive today?	☐ COVID vaccine<br>☐ FLU Vaccine<br>☐ Tetanus Vaccine<br>☐ Other

© 2025 Molina MF et al. *JAMA Network Open*.

Specify other	______________________________
IF NO to not receiving ANY vaccines, give a reason:	◯ Ordered but not administered / ne se administró<br>◯ Provider did not order / El proveedor no lo solicitó<br>◯ No vaccine available / No hay vacuna disponible<br>◯ Patient was ineligible because of illness / El paciente no era elegible debido a una<br>◯ efermedad Patient refused / el paciente se negó<br>◯ Control, patient did not ask for vaccine<br>◯ Other / otro<br>◯ Attempted but unable to contact provider
Specify other	______________________________

FOR CRC ONLY

Please complete this section after enrollment has been completed.

Did the patient consent to a follow up, please double check and confirm.<br>up	Patient consented to follow up<br>◯ Patient did not consent to follow<br>◯
Did the participant agree to a phone follow up?	◯ Yes<br>◯ Yes, but patient does not have<br>◯ phone No
Recorded relevant fields in Follow up Excel sheet	☐ Name<br>☐ Study<br>ID ☐<br>MRN<br>☐ Phone Number
If the patient was not able to complete the second survey, give a reason.	◯ Patient left AMA<br>◯ Patient was discharged<br>◯ Patient refused survey<br>◯ Withdrawn<br>◯ Other
Specify other	______________________________

© 2025 Molina MF et al. *JAMA Network Open*.

eFigure. Study Flow and Procedures

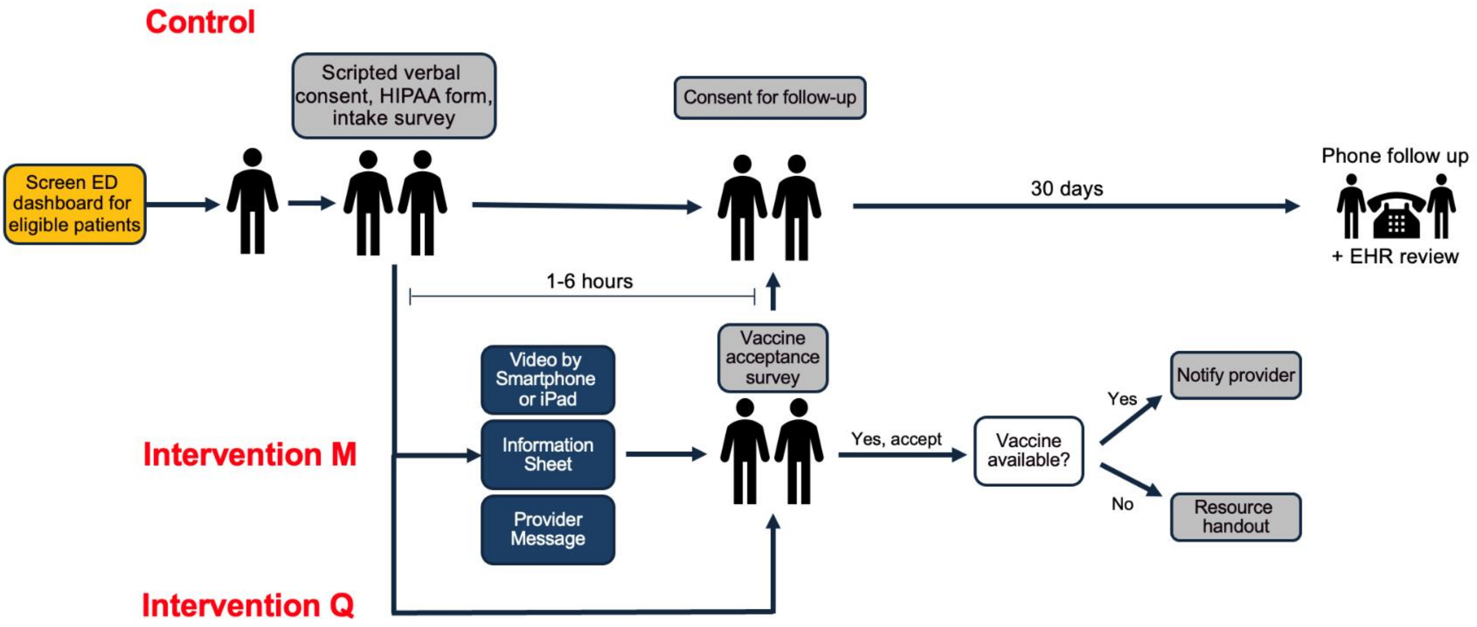

© 2025 Molina MF et al. *JAMA Network Open*.

eTable 2. Primary and Secondary Outcomes, Stratified by Vaccine Availability

eTable 2a. Primary and Secondary Outcomes

	Proportion			Difference in Proportion		
	Control	Intervention M	Intervention Q	Int M v. Control	Int Q v. Control	Int Q v. Int M
**Vaccinated in 30 days**	10 (3.0%)	14 (5.7%)	11 (4.0%)	2.7% (-0.8% to 6.3%)	1.0% (-2.0% to 4.2%)	-1.6% (-5.5% to 2.2%)
**Accepted in ED**	0 (0.0%)	73 (29.6%)	90 (33.0%)	29.6% (23.7% to 35.1%)	33.0% (27.2% to 38.4%)	3.4% (-4.6% to 11.3%)
**Accepted in ED after cost consideration**	0 (0.0%)	61 (24.7%)	68 (24.9%)	24.7% (19.2% to 30.0%)	24.9% (19.6% to 29.9%)	0.2% (-7.2% to 7.6%)
**Vaccinated in the ED**	0 (0.0%)	8 (3.2%)	7 (2.6%)	3.2% (0.9% to 5.7%)	2.6% (0.5% to 4.7%)	-0.7% (-3.8% to 2.3%)

eTable 2b. Primary and Secondary Outcomes (Sites with Vaccine Available)

	Proportion			Difference in Proportion		
	Control	Intervention M	Intervention Q	Int M v. Control	Int Q v. Control	Int Q v. Int M
**Vaccinated in 30 days**	5 (3.3%)	13 (9.8%)	8 (6.2%)	6.5% (0.5% to 12.5%)	2.8% (-2.4% to 8.2%)	-3.7% (-10.4% to 3.1%)
**Accepted in ED**	0 (0.0%)	39 (29.5%)	42 (32.3%)	29.5% (21.3% to 37.0%)	32.3% (23.8% to 40.0%)	2.8% (-8.4% to 13.9%)
**Accepted in ED after cost consideration**	0 (0.0%)	30 (22.7%)	25 (19.2%)	22.7% (15.2% to 29.7%)	19.2% (12.1% to 25.9%)	-3.5% (-13.3% to 6.4%)
**Vaccinated in the ED**	0 (0.0%)	8 (6.1%)	7 (5.4%)	6.1% (1.6% to 10.5%)	5.4% (1.1% to 9.7%)	-0.7% (-6.5% to 5.2%)

eTable 2c. Primary and Secondary Outcomes (Sites without Vaccine Available)

	Proportion			Difference in Proportion		
	Control	Intervention M	Intervention Q	Int M v. Control	Int Q v. Control	Int Q v. Int M
**Vaccinated in 30 days**	5 (2.7%)	1 (0.9%)	3 (2.1%)	-1.9% (-5.0% to 1.9%)	-0.6% (-4.2% to 3.2%)	1.2% (-2.5% to 4.6%)
**Accepted in ED**	0 (0.0%)	34 (29.6%)	48 (33.6%)	29.6% (21.0% to 37.7%)	33.6% (25.5% to 41.0%)	4.0% (-7.4% to 15.2%)
**Accepted in ED after cost consideration**	0 (0.0%)	31 (27.0%)	43 (30.1%)	27.0% (18.7% to 35.0%)	30.1% (22.2% to 37.4%)	3.1% (-8.0% to 14.0%)
**Vaccinated in the ED**	0 (0.0%)	0 (0.0%)	0 (0.0%)	0.0% (-1.7% to 2.3%)	0.0% (-1.6% to 1.9%)	0.0% (-2.3% to 2.0%)

© 2025 Molina MF et al. *JAMA Network Open*.
