# Supplementary material for: COVID-19 Booster Vaccine Messaging in Emergency Departments: A Cluster Randomized Clinical Trial
Source: JAMA Netw Open. 2025 Oct 15;8(10):e2537655. doi: 10.1001/jamanetworkopen.2025.37655 (PMC12529187; doi:10.1001/jamanetworkopen.2025.37655)
Supplement: Supplement 3. — Data Sharing Statement [file jamanetwopen-e2537655-s003.pdf]

Data Sharing Statement

Molina. COVID-19 Booster Vaccine Messaging in Emergency Departments. *JAMA Netw Open*. Published October 15, 2025. doi:10.1001/jamanetworkopen.2025.37655

Data

Additional Information: ClinicalTrials.gov Identifier: NCT06156215 (https://clinicaltrials.gov/study/NCT06156215)
Data available: No

Additional Information

Explanation for why data not available: Data can be made available on a case-by-case basis upon request and after consideration by study investigators.
